# The Origin and Evolutionary History of HIV-1 Subtype C in Senegal

**DOI:** 10.1371/journal.pone.0033579

**Published:** 2012-03-28

**Authors:** Matthieu Jung, Nafissatou Leye, Nicole Vidal, Denis Fargette, Halimatou Diop, Coumba Toure Kane, Olivier Gascuel, Martine Peeters

**Affiliations:** 1 UMI233, TransVIHMI, IRD (Institut de Recherche pour le Développement) and University of Montpellier 1, Montpellier, France; 2 UMR 5506, Méthodes et Algorithmes pour la Bioinformatique, Laboratoire d'Informatique, de Robotique et de Microélectronique de Montpellier, CNRS and University of Montpellier 2, Montpellier, France; 3 Laboratory of Bacteriology and Virology, Le Dantec University Teaching Hospital, Dakar, Senegal; 4 UMR RPB, Institut de Recherche pour le Développement, La Recherche Agronomique pour le Développement and University of Montpellier 2, Montpellier, France; Jiangsu University, China

## Abstract

**Background:**

The classification of HIV-1 strains in subtypes and Circulating Recombinant Forms (CRFs) has helped in tracking the course of the HIV pandemic. In Senegal, which is located at the tip of West Africa, CRF02_AG predominates in the general population and Female Sex Workers (FSWs). In contrast, 40% of Men having Sex with Men (MSM) in Senegal are infected with subtype C. In this study we analyzed the geographical origins and introduction dates of HIV-1 C in Senegal in order to better understand the evolutionary history of this subtype, which predominates today in the MSM population

**Methodology/Principal Findings:**

We used a combination of phylogenetic analyses and a Bayesian coalescent-based approach, to study the phylogenetic relationships in *pol* of 56 subtype C isolates from Senegal with 3,025 subtype C strains that were sampled worldwide. Our analysis shows a significantly well supported cluster which contains all subtype C strains that circulate among MSM in Senegal. The MSM cluster and other strains from Senegal are widely dispersed among the different subclusters of African HIV-1 C strains, suggesting multiple introductions of subtype C in Senegal from many different southern and east African countries. More detailed analyses show that HIV-1 C strains from MSM are more closely related to those from southern Africa. The estimated date of the MRCA of subtype C in the MSM population in Senegal is estimated to be in the early 80's.

**Conclusions/Significance:**

Our evolutionary reconstructions suggest that multiple subtype C viruses with a common ancestor originating in the early 1970s entered Senegal. There was only one efficient spread in the MSM population, which most likely resulted from a single introduction, underlining the importance of high-risk behavior in spread of viruses.

## Introduction

HIV-1 group M, which predominates in the global HIV/AIDS epidemic, can be further subdivided into subtypes (A–D, F–H, J, K), sub-subtypes (A1 to A4, F1 and F2), circulating recombinant forms (CRF01 to CRF51) and numerous unique recombinant forms (URFs) (www.hiv.lanl.gov). This genetic diversity has an impact on almost all aspects of the management of this infection going from identification and monitoring of infected persons, to treatment efficacy and vaccine design [1]–[3]. The classification of HIV strains has also helped in tracking the course of the HIV pandemic [4]. Numerous molecular epidemiological studies showed a heterogeneous geographic distribution of the different HIV-1 M subtypes and CRFs. The initial diversification of group M most likely occurred within or near the Democratic Republic of Congo (DRC) [5], [6], where the highest diversity of group M strains has been observed and the earliest cases of HIV-1 infection (1959 and 1960) have been documented in Kinshasa, the capital city [7]. Different HIV variants have then spread across the world, and the epidemics in the different continents and countries are the result of different founder effects. Today, subtype C accounts for 50% of all infections [8]. The majority of subtype C infections are found in southern Africa where they represent almost 100% of circulating HIV-1 strains. Subtype C also predominates in India, Ethiopia and southern China, and has entered East Africa, Brazil, and many European countries. With increasing mobility and human migration, HIV-1 variants inevitably intermix in different parts of the world and the distribution of the different HIV-1 variants is a dynamic process.

In Senegal, which is located at the tip of West Africa, both AIDS viruses, HIV-1 and HIV-2, co-circulate. HIV-2 was first described in Senegal, but like in other West African countries, the prevalence of HIV-2 remained low and is decreasing [9], [10]. Today HIV-1 predominates and since the description of the first HIV-1 AIDS case in 1986, HIV-1 seroprevalence remains below 1% in the general population but can reach up to 20% in population groups with high risk behavior like female sex workers (FSWs) or men having sex with men (MSM) [11]. Several studies showed that CRF02_AG predominates in Senegal, representing 50–70% of circulating strains in the general population and FSWs, but in contrast to surrounding west African countries, a wide diversity of other HIV-1 variants co-circulate; subtypes A1, A3, B, D, F, G, H, CRF01, CRF06, CRF09, CRF11, CRF45 and HIV-1 group O have all been documented [10], [12]–[14]. As mentioned above, the distribution of HIV-1 subtypes/CRFs can differ between geographic origins and between population groups. Recently our studies showed that 40% of MSM in Senegal are infected with subtype C, which is in strong contrast with 4% to 10% in the general population and FSWs [10], [12]–[15]. The factors associated with the rapid spread of subtype C and its predominance in the global epidemic are not entirely known, but in certain regions where it has been introduced, subtype C has overtaken other HIV-1 variants [16]. The high prevalence and the rapid spread of subtype C among MSM needs thus particular attention because this could also lead to an increase overtime of subtype C in the general population because more than 90% of MSM recognize having sex with women [17].

Using a combination of phylogenetic analyses and a Bayesian coalescent-based approach, we studied the phylogenetic relationships of subtype C isolates from Senegal with other subtype C strains that were sampled worldwide, in order to define the origin and onset of the subtype C epidemic in MSM in Senegal.

## Results

### Origin of subtype C sequences in Senegal

Among the HIV-1 subtype C pol sequences that were downloaded, we first eliminated all sequences that were not identified as subtype C (i.e. intersubtype recombinants) by the REGA-subtyping tool and kept only one isolate per patient. The final dataset includes a total of 3,081 sequences spanning a 1,011 bp fragment in pol between positions 2,253 and 3,263 on the HXB2 genome, including 56 (among which 24 MSM and 18 newly sequenced) strains from Senegal (Table 1 and Table S1). Sequences were included from 4 different continents and 61 countries: Africa (22 countries), the Americas (7 countries), Asia (9 countries) and Europe (23 countries) (Table 2). The majority (67.73%) of the sequences are from Africa and more precisely from southern Africa (55.14%) that is South Africa (22.36%) and Zambia (20.55%), and to a lower extent Botswana (4.32%), Mozambique (3.18%), Malawi (2.30%), Swaziland (1.53%), and Zimbabwe (0.91%). Subtype C sequences from Asia are predominantly from India (355 sequences on a total of 380) and those from the Americas mainly from southern Brazil (253 sequences on a total of 299). Subtype C sequences from Europe represent 10.22% of the dataset and are collected from 23 different countries, without a single country or area that predominates in the dataset.

**Table 1:** HIV-1 subtype C strains from Senegal included in this study.

Strain identification	Accession Number	Year of isolation	Population group	Reference
90SN-90SE364	AY713416	1990	general population	[53]
98SN-66HPD	AJ583722	1998	general population	[54]
99SN-159HALD	AJ583716	1999	general population	[54]
99SN-142HPD	AJ583715	1999	general population	[54]
98SN-39HALD	AJ287005	1998	general population	[55]
99SN-86HPD	AJ583739	1999	general population	[54]
04SN-MS003	FM210753	2004	MSM	[15]
04SN-MS883	FM210752	2004	MSM	[15]
04SN-MS855	FM210749	2004	MSM	[15]
04SN-MS835	FM210745	2004	MSM	[15]
04SN-MS821	FM210741	2004	MSM	[15]
04SN-MS816	FM210740	2004	MSM	[15]
04SN-MS779	FM210737	2004	MSM	[15]
04SN-MS700	FM210736	2004	MSM	[15]
04SN-MS540	FM210726	2004	MSM	[15]
04SN-MS522	FM210725	2004	MSM	[15]
04SN-MS492	FM210723	2004	MSM	[15]
04SN-MS048	FM210722	2004	MSM	[15]
04SN-MS481	FM210718	2004	MSM	[15]
04SN-MS477	FM210717	2004	MSM	[15]
04SN-MS475	FM210716	2004	MSM	[15]
04SN-MS448	FM210712	2004	MSM	[15]
04SN-MS422	FM210709	2004	MSM	[15]
04SN-MS245	FM210699	2004	MSM	[15]
04SN-MS029	FM210691	2004	MSM	[15]
04SN-MS015	FM210689	2004	MSM	[15]
04SN-MS011	FM210687	2004	MSM	[15]
04SN-MS010	FM210686	2004	MSM	[15]
04SN-MS007	FM210685	2004	MSM	[15]
04SN-MS002	FM210684	2004	MSM	[15]
03SN-980HALD	FN599776	2003	general population	[14]
03SN-965HALD	FN599773	2003	general population	[14]
02SN-510HALD	FN599737	2002	general population	[14]
99SN-67HDP	FN599718	1999	general population	[14]
09SN-SNA3-366	HM002544	2009	not known	unpublished
08SN-SNA3-220	HM002517	2008	not known	unpublished
08SN-SNA3-191	HM002515	2008	not known	unpublished
07SN-SNA3-107	HM002507	2007	not known	unpublished
02SN-260HALD	HE588158	2002	general population	this study
03SN-154HALD	HE588157	2003	general population	this study
03SN-321HALD	HE588156	2003	general population	this study
03SN-L065	HE588149	2003	general population	this study
06SN-463HALD	HE588155	2006	general population	this study
07SN-2658HALD	HE588150	2007	general population	this study
07SN-2909HALD	HE588151	2007	general population	this study
07SN-2911HALD	HE588152	2007	general population	this study
07SN-2936HALD	HE588153	2007	general population	this study
07SN-3076HALD	HE588154	2007	general population	this study
00SN-102HALD	HE588159	2000	general population	this study
97SN-1119	HE588162	1997	general population	this study
02SN-478HALD	HE588163	2002	general population	this study
97SN-14Fann	HE588165	1997	general population	this study
97SN-25Fann	HE588164	1997	general population	this study
96SN-1083	HE588166	1996	general population	this study
97SN-1186	HE588161	1997	general population	this study
97SN-1189	HE588160	1997	general population	this study

**Table 2:** Numbers of HIV-1 subtype C strains from different countries that were included in this study.

Continent	Country	Number	%
***Africa***		***2087***	***67.73***
	Botswana	133	4.32
	Burundi	91	2.95
	Democratic Republic of Congo	19	0.62
	Djibouti	1	0.03
	Equatorial Guinea	1	0.03
	Eritrea	2	0.06
	Ethiopia	99	3.21
	Gabon	1	0.03
	Kenya	4	0.13
	Malawi	71	2.30
	Mali	1	0.03
	Mozambique	98	3.18
	Niger	4	0.13
	Senegal	56	1.82
	Somalia	1	0.03
	South Africa	689	22.36
	Sudan	10	0.32
	Swaziland	47	1.53
	Tanzania	82	2.66
	Uganda	16	0.52
	Zambia	633	20.55
	Zimbabwe	28	0.91
***America***		***299***	***9.71***
	Argentina	8	0.26
	Brazil	253	8.21
	Cuba	25	0.81
	Honduras	1	0.03
	United States of America	9	0.29
	Uruguay	2	0.06
	Venezuela	1	0.03
***Asia***		***380***	***12.33***
	China	7	0.23
	India	355	11.52
	Israël	5	0.16
	Myanmar	1	0.03
	Philippines	1	0.03
	Russia	1	0.03
	South Korea	2	0.06
	Taiwan	1	0.03
	Yemen	7	0.23
***Europe***		***315***	***10.22***
	Austria	3	0.10
	Belgium	35	1.14
	Cyprus	8	0.26
	Czech Republic	11	0.36
	Danmark	21	0.68
	Finland	6	0.19
	France	7	0.23
	Georgia	1	0.03
	Germany	7	0.23
	Greece	3	0.10
	Italy	22	0.71
	Luxemburg	3	0.10
	Norway	16	0.52
	Poland	2	0.06
	Portugal	28	0.91
	Roumania	35	1.14
	Slovakia	1	0.03
	Spain	26	0.84
	Sweden	64	2.08
	Switzerland	2	0.06
	The Netherlands	8	0.26
	Ukraine	3	0.10
	United Kingdom	3	0.10
***Total***		***3081***	

**Figure 1:**
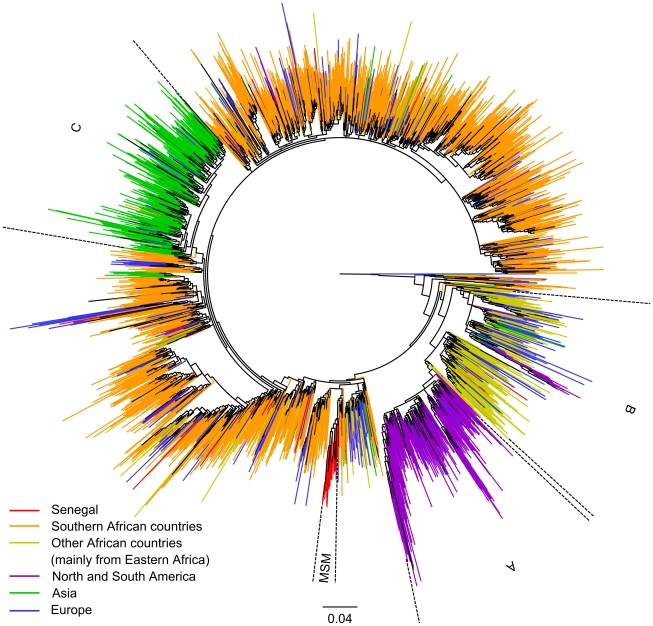
Maximum likelihood phylogenetic tree based on 3,081 HIV-1 subtype C *pol* sequences. Maximum likelihood (PhyML) phylogenetic tree based on 1,011 nucleotide sites of *pol* gene sequence (nucleotides 2,253–3,263 of HXB2 coordinates) from 3,081 HIV-1 subtype C isolates. Sequences were isolated in the countries shown in Table 2. Sequences are colored to their region of origin: Senegal in red, Southern African countries (South-Africa, Botswana, Malawi, Mozambique, Swaziland, Zambia and Zimbabwe) in orange, other African countries (mainly from the East) in yellow, North and South America in purple, Asia in green and Europe in blue. The branch support (aLRT) of clade A, B, C and MSM are of 73%, 76%, 82% and 99% respectively.

The maximum likelihood (PhyML) tree of the 3,081 subtype C sequences is shown in Figure 1. The strains from Senegal are highlighted in red, those from southern Africa (South Africa, Zambia, Zimbabwe, Malawi, Mozambique, Botswana, and Swaziland) in orange and those from the other African countries, which are predominantly from East Africa, in yellow. Strains from Asia, the Americas, and Europe are highlighted in green, purple and blue respectively. The sequences from Senegal are interspersed with the other African strains, but one significant cluster (98.9% aLRT support), which comprised all sequences obtained from MSM from Senegal, was identified. The phylogenetic tree shows also separate clades for subtype C strains from southern Africa and one from eastern Africa (cluster B, 75.9% aLRT support), each of which contains sequences from Senegal. The tree shows the presence of two other major clusters, one for the majority of South American (cluster A, purple) and one for the Asian strains (cluster C, green), each apparently resulting from different single introductions, but no strain from Senegal was observed in these clusters. The clusters from South America and Asia are each supported by 72.7% and 82.3% aLRT values, respectively. No significant cluster of European subtype C was observed, they are all interspersed with strains from different geographic origins mainly in Africa and in Asia and southern America. In order to exclude the possibility of artifactual phylogenetic clustering due to drug induced convergent evolution, especially for the clades from Senegal, the phylogenetic tree analysis was repeated on an alignment where 43 (i.e. 129 nt, ∼12.7% of the full alignment) codon positions known to be associated with major resistance mutations were removed. This analysis shows the same subtype C clusters (Figure S1).

**Figure 2:**
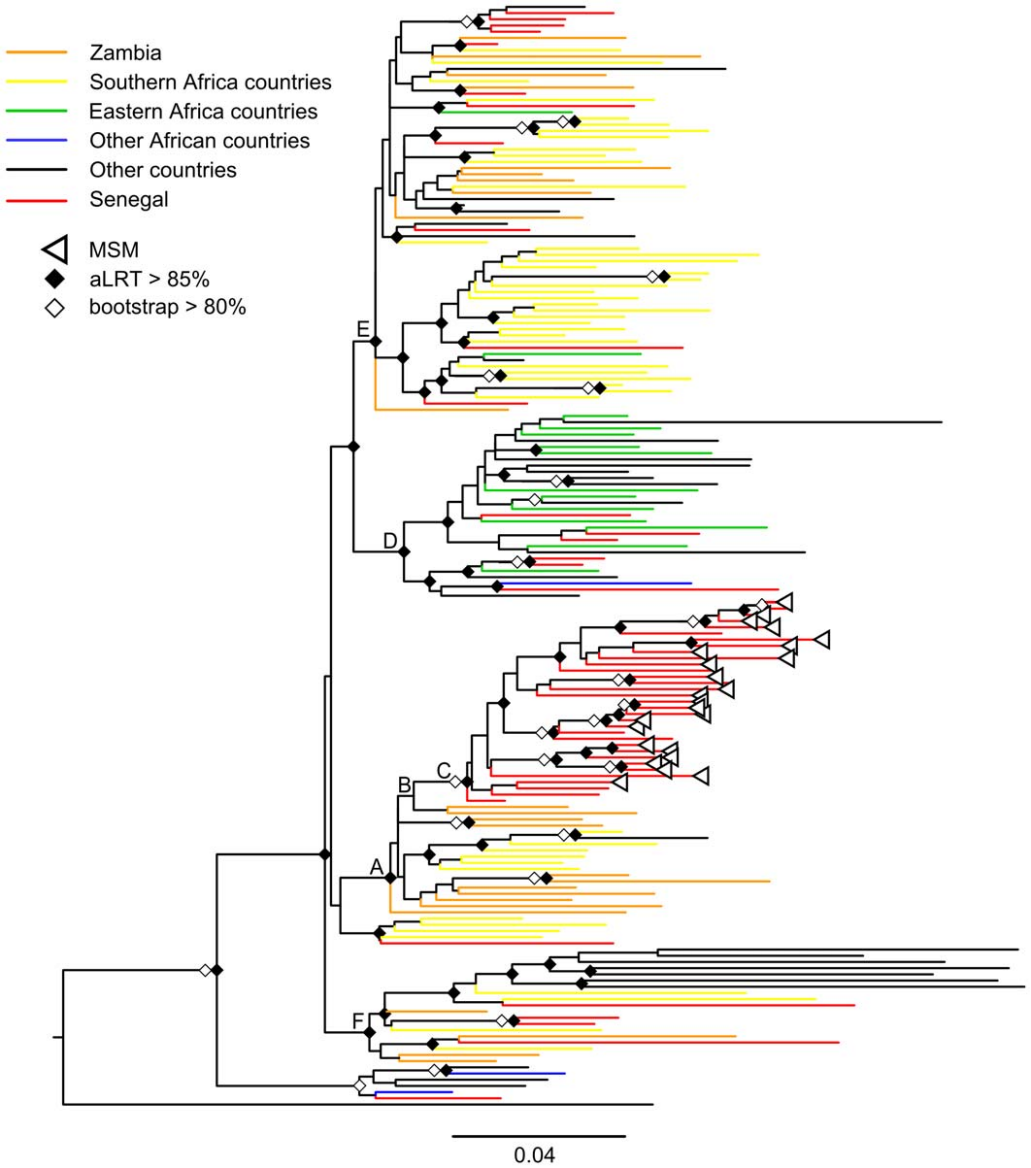
Maximum likelihood phylogenetic tree constructed from 56 HIV-1 C *pol* sequences from Senegal and 121 close relatives. Detailed maximum likelihood (PhyML) phylogenetic tree constructed using 1,011 nucleotide sites of *pol* gene sequence (nucleotides 2,253–3,263 of HXB2 coordinates) from 177 HIV-1 subtype C isolates from Senegal and close relatives (see text). Branch support values (bootstrap and aLRT) are displayed (see figure legend). Colors indicate the geographic origin and sequences were isolated in the following countries: 56 in red from Senegal, 25 in orange from Zambia, 49 in yellow from southern Africa (Botswana 6; Mozambique 5; Swaziland 2; South Africa 35; Zimbabwe 1), 12 in green from East Africa (Burundi 2; Ethiopia 9; Kenya 1; Sudan 2), 3 in blue from other African countries (DRC 1; Equatorial Guinea 1; Gabon 1) and 30 in black from European and Asian countries (Belgium 4; China 1; Germany 2; Denmark 1; Spain 5; France 1; Greece 1; Israel 1; India 1; Italia 1; Luxembourg 1; Norway 2; Portugal 2; Sweden 7).

The above analysis showed that subtype C was introduced into Senegal at multiple occasions. Figure 2 shows in more details the subtype C sequences that are most closely related to those observed in Senegal. As described in Materials and Methods, only sequences that branched with one or more sequences from Senegal until the second ancestral node in the phylogenetic tree of the 3,081 sequences, were used for this subtree. In addition to the 56 sequences from Senegal, 121 other subtype C sequences were included (Table S2), representing 5.7% of the total alignment. Figure 2 shows the tree obtained by PhyML with strains colored according to their geographic origin (the same tree with strain names is available in Figure S2). HIV-1 strains from Zambia are represented by a separate color in this tree because strains from this country are frequently present. The majority of the subtype C strains from Senegal and those from the MSM cluster (node C) are falling in clusters (aLRT >85%) which are mainly represented by strains from Zambia and other countries from southern Africa (for example node A, E and F). Nevertheless, some strains from Senegal are related to subtype C from east African countries (majority Ethiopia: node D). Although the exact country at the origin of the most recent common ancestor of the MSM strains remains uncertain, this was most likely in southern Africa. The first ancestral node to the MSM cluster (node B) suggests an origin in Zambia, but this node is only supported with 83.7% aLRT and 11% bootstrap values. The first ancestral node (node A), supported by an aLRT value of 94.7% and a bootstrap value of 49%, contains mainly strains from Zambia but also from other southern African countries. The Bayesian phylogenetic tree analysis performed with MrBayes shows similar results (Figure S3).

### Dating the subtype C epidemic in Senegal and MSM population

**Figure 3:**
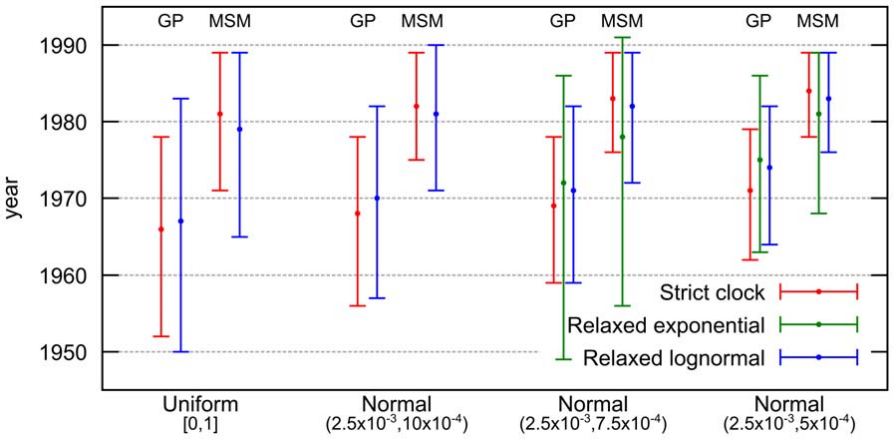
Dating the subtype C epidemic in general and MSM populations in Senegal. Coalescent based estimations (BEAST) and 95% highest posterior density (HPD) intervals of the MRCA dates of 56 HIV-1 subtype C *pol* sequences obtained from the general and the MSM population. Results are displayed for all tested substitution rate priors and molecular clock models, except for relaxed exponential with both less informative priors which provides very large 95% HPD intervals and shows convergence problems (see Table S3 for detailed results, including substitution rate estimations).

We used a Bayesian MCMC approach implemented in BEASTv1.6.1 to estimate the dates of the most recent common ancestors (MRCAs) for the subtype C sequences from Senegal in the general population and for the subtype C epidemic in the MSM population. We used the Bayesian skyride population growth model associated to three molecular clock models: strict, relaxed uncorrelated lognormal, and relaxed uncorrelated exponential. Moreover, we used four different priors on the average substitution rate among branches with varying informative levels. Figure 3 shows the resulting estimations of the MRCA dates for the different models and priors used. More details are provided in Table S3, including substitution rate estimations.

Bayes factors (BF) indicate that the relaxed exponential model has a small advantage (BF in the 3 to 5 range) over the relaxed lognormal model, which in turn is slightly better (BF in the 3 to 6 range) than the strict molecular clock. However, the relaxed exponential model becomes non-informative when non- or poorly informative priors on the substitution rate are used (U[0,1] and N[2.5×10^−3^, 10×10^−4^], see Materials and Methods), which reveals spurious peaks leading to very large (up to ∼400 years) 95% Highest Posterior Density (HPD) intervals and unrealistic estimates. Except in these two cases, the results with all models and priors are quite consistent. As expected, when we used more informative priors we obtained more restricted 95% HPD intervals. Nevertheless, the median date estimates of the MRCAs of subtype C in the general population of Senegal and for the MSM cluster are similar for all models and priors, indicating likely epidemic origins in the early 80's, in the MSM population. The MRCA for the subtype C strains that entered at multiple occasions into the general population (i.e. heterosexual or mother to child transmission), is estimated in the early 70's.

**Figure 4:**
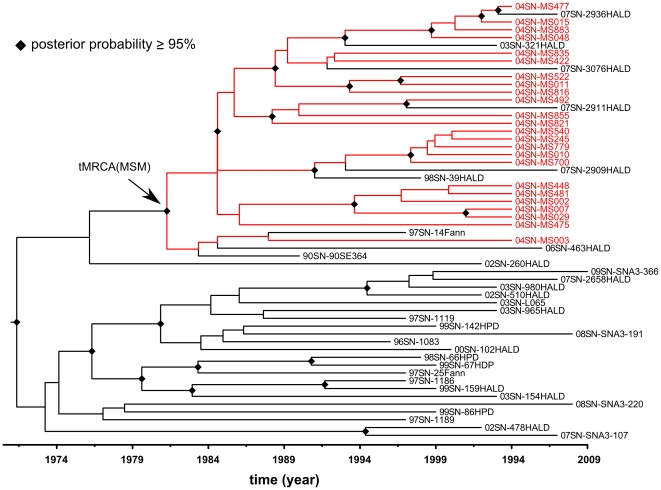
Bayesian tree with timescale of 56 HIV-1 C *pol* sequences from Senegal. Maximum clade credibility tree with time scale obtained with BEAST using 1,011 nucleotide sites of *pol* gene sequences (nucleotides 2,253–3,263 of HXB2 coordinates) from 56 HIV-1 subtype C isolates from Senegal. This tree is obtained using the relaxed uncorrelated lognormal molecular clock model and moderately informative substitution rate prior (Normal: 2.5×10^−3^,7.5×10^−4^). Clades with posterior probabilities ≥95% are indicated by diamonds. MSM isolates are colored in red.

To illustrate in more detail the MRCA of the subtype C strains in the MSM population and their relation to the other HIV-1 C strains from Senegal, the maximum clade credibility (MCC) tree with time scale obtained from BEAST is shown in Figure 4. We see the same MSM cluster as in the phylogeny of Figure 2 (see also Figure S2 and S3), and the early 70's and 80's dates for the MRCAs of general and MSM population respectively.

We verified whether presence of drug resistance mutations could have an impact on MRCA dates and substitution rate estimations. Therefore calculations were repeated on the three different molecular clock models and for the four priors on an alignment where 43 codon positions known to be associated with major resistance mutations were removed. This analysis showed no significant difference, compared to the results obtained with the complete alignment (Table S3 for details on estimations and Figure S4 for the MCC tree with time scale).

**Figure 5:**
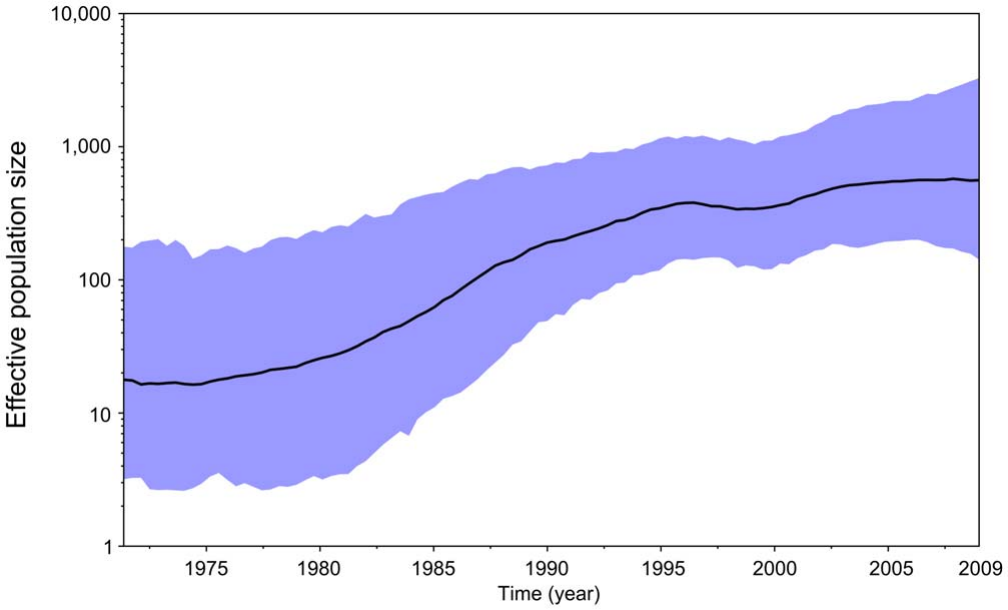
Bayesian skyride plot of HIV-1 C demographic growth in Senegal using 56 *pol* sequences. Estimates of HIV-1 C effective number of infections (*N_e_*) over time from 56 Senegalese *pol* sequences using a Bayesian skyride plot in BEAST with relaxed uncorrelated lognormal molecular clock and moderately informative substitution rate prior (ucld.mean Normal: 2.5×10^−3^, 7.5×10^−4^). The X-axis represents the time in year. The Y-axis represents the HIV-1 effective number of infections (log_10_ scale). The black line marks the median estimate for *N_e_* and the blue shadow region displays the 95% highest posterior density (HPD) interval.

Finally, our reconstruction of the demographic history of HIV-1 C in Senegal identified an initial, slow growth phase until the end of the 70's followed by a period of quick exponential-like growth at the end of the 90's where the epidemic growth became slower (Figure 5).

## Discussion

In this study we analyzed the geographical origins and introduction dates of HIV-1 subtype C in Senegal in order to better understand the evolutionary history of this subtype which predominates today in the MSM population [15]. Our evolutionary reconstructions suggest that multiple subtype C viruses with a common ancestor originating in the early 1970s entered the country, followed by a sharp growth of the effective number of infections over the next decade.

This analysis of more than 3,000 globally collected reference sequences most likely provides an adequate representation of global subtype C diversity, and provides also additional information on the subtype C epidemic in other continents. The phylogenetic tree analysis showed several major clusters of subtype C sequences, mainly related to the continent of origin, like Asia, Southern America or Africa, except for Europe. Interestingly, among the African strains, a separate cluster of strains derived from patients living in east African countries was observed [18], and subtype C strains from Europe do not form a separate cluster and are interspersed among the different continents and major clusters. Our data also confirm the previously reported link of the subtype C epidemic in Brazil with east Africa [19]–[22].

Our analyses with various methods (PhyML, MrBayes and BEAST) showed a significantly well-supported cluster which contained all subtype C strains that circulate among MSM in Senegal. The MSM cluster and other strains from Senegal are widely dispersed among the different subclusters of African strains, suggesting multiple introductions of subtype C into Senegal from many different southern and also eastern African countries. More detailed analyses showed that the majority of the HIV-1 C strains from Senegal, including those circulating among MSM, are more closely related to strains from southern African countries, mainly Zambia. The cluster of subtype C strains derived from the MSM population includes also strains from HIV-1 infected men from Senegal, who were not identified as MSM. Homosexuality is illegal in Senegal and male-to-male sex is condemned by political and religious authorities and by the general population, therefore most MSM keep their sexual life secret, including from their own family and more than 90% of MSM reported having sex also with women [17]. Thus, these additional strains in the MSM cluster are most likely from individuals with male-to-male sex activities. Subtype C in MSM may have its origin directly from southern Africa but it is also possible that the ancestor of this subtype C cluster circulated already for a certain period in the general population in Senegal before it was introduced into the MSM group.

The wide diversity and multiple introductions of subtype C fit also with the distribution of the HIV-1 variants in the general population in Senegal. Several studies showed that in addition to CRF02_AG, many other HIV-1 subtypes and CRFs are also present in the country, reflecting multiple introductions [10], [12]–[14]. This is most likely related to the important trading activity and travel links of the country with many other African countries [23], [24]. Our estimates suggest that the MRCA of the subtype C strains that entered Senegal was in the early 1970's, about 10–15 years before the description of the first HIV-1 AIDS case in the country or the first HIV-1 subtype C strain in 1988 in Senegal [25]. The MRCA date estimate of subtype C in Senegal is relatively close to those estimated in other African countries, like 1966 for subtype C in Ethiopia [26], beginning of the 70's for Zimbabwe [27] or in the late 60's for Malawi [28]. As expected, we found that MRCA of subtype C in Senegal is not specific, because multiple introductions occurred, and our MRCA date estimate corresponds most likely to those of subtype C strains outside Senegal. In contrast to southern African countries, subtype C did not become the predominant strain in Senegal and did only spread efficiently in the MSM population, underlining the importance of high risk behavior in spread of viruses [29]. The MRCA of subtype C in the MSM population is estimated in the early 80's and is the result of a single introduction. This estimate coincides with the period where the HIV-1 C epidemic started a quick exponential-like growth phase in Senegal for nearly 15 years according to the Bayesian skyride analysis.

Our study showed also that analysis of alignments with or without codons that are associated with drug resistance did not have a significant impact on phylogenetic clustering or on MRCA date and substitution rate estimations. Among the different molecular clock models used, Bayes factors suggested the use of the relaxed exponential molecular clock above the most frequently used relaxed lognormal molecular clock. However, the very large confidence intervals and convergence problems with the exponential model with poorly informative priors, and the almost similar results with informative priors for both models are probably at the basis for the preferential use of the relaxed lognormal molecular clock model for HIV.

Previous studies suggest that subtype C could spread more efficiently due to the predominance of CCR5 variants or a stronger predisposition for localization in the female genital mucosa than other subtypes, which may facilitate both vertical and heterosexual transmission [30]–[33]. Increase of subtype C could also have implications on treatment because other subtype C specific mutations have been documented and commercial drug resistance assays cannot correctly test subtype C infections [2], [34]–[36]. A cross-sectional study of women in Kenya indicated that women infected with subtype C had a higher viral load and lower CD4 counts than those infected with subtypes A and D, which could also have an impact on pathogenesis and transmission [37]. Therefore, it is important to continue to monitor HIV-1 subtype/CRF distribution among different population groups in Senegal. However, in order to be able to compare trends over time, such studies should be organized in a standardized way. For example, WHO proposed standardized protocols for surveillance of drug resistance mutations in recently infected individuals [38]. These studies can be combined with subtype/CRF characterization.

Because MSM reported having sex also with women, they could potentially serve as a bridge between high-risk men and low-risk women. This sexual mixing pattern might contribute in the future to the subsequent increase of subtype C in the general population. An increase from 4% in 2000 to almost 10% between 2000 and 2010 among the general population in Senegal has already been observed, and subtype C sequences recently obtained from HIV-1 C infected women in 2011 that cluster within the clade of strains from the MSM population have now been observed (Coumba Toure Kane, unpublished results). Understanding the origins and dispersal patterns of HIV-1 clades at regional and country levels is useful to improve the characterization and control of HIV spread. Continuous monitoring of HIV variants seems necessary to adapt treatment and vaccine strategies to be efficient against local and contemporary circulating HIV variants.

## Materials and Methods

### Nucleotide sequence dataset

In order to increase the number of sequences and to cover a wide geographic range, we used the *pol* region for our analysis. *Pol* sequences are highly studied because they are the target of antiretroviral drugs. A total of 56 subtype C *pol* gene sequences from Senegal were used in this study. Thirty-eight were obtained from the Los Alamos HIV sequence database (www.hiv.lanl.gov) from previously published reports and eighteen were newly characterized from ongoing molecular epidemiology and/or drug resistance studies mainly in Dakar, the capital city of Senegal (Table 1). We downloaded only sequences that were at least 1,000 nucleotides in length and spanning the genomic region which covers protease and majority of RT in *pol* between positions 2,253–3,263 on the HXB2 genome. Sequences were from blood samples collected between 1990 and 2009. In addition, all available subtype C sequences spanning the same genomic region and for which country of origin and sampling year were known, were also downloaded from the Los Alamos HIV database (www.hiv.lanl.gov). We then submitted all the sequences to the REGA subtyping tool v.2 to confirm subtype assignments and to eliminate eventual intersubtype recombinants [39], [40]. We selected one sequence per individual when sequential sequences were available or when sequences were epidemiologically linked by direct donor–recipient transmission.

### HIV-1 pol sequencing

The 18 new HIV-1 *pol* sequences were obtained with an in-house technique as previously described [41]. Briefly, RNA was extracted using the QIAamp Viral RNA extraction kit (Qiagen SA, Courtabeauf, France) and processed for reverse transcription polymerase chain reaction (RT-PCR) with the integrase specific primer IN3 5′-TCTATBCCATCTAAAAATAGTACTTTCCTGATTCC-3′ using the Expand reverse transcriptase (Roche Diagnostics, Meylan, France) according to the manufacturer's instructions. The resulting cDNA served as template in the subsequent nested PCR reaction during which a 1,865 base pairs fragment, corresponding to the protease and the first 440 amino acids of the reverse transcriptase region of the *pol* gene, was amplified with previously described primers and cycling conditions using the Expand Long Template PCR system (Roche Diagnostics, Meylan, France). The amplified HIV-1 nucleic acid fragments were purified using the Geneclean Turbo Kit (Q-Biogen, MPbiomedicals, France) and directly sequenced with primers encompassing the *pol* region using BigDye Terminator version 3.1 (Applied Biosystems, Courtaboeuf, France) according to the manufacturer's instructions. Electrophoresis and data collection were done on an Applied Biosystems 3130XL Genetic Analyzer. The sequenced fragments from both strands were reconstituted using Seqman II from the DNAstar package v5.08 (Lasergene, Madison, WI, USA).

### Sequence alignment and phylogenetic tree analysis

The 18 newly obtained sequences were aligned with the alignment of subtype C sequences downloaded from the Los Alamos HIV database, using the L-INS-i method from MAFFT [42], [43], and then manually edited with MEGA5 [44]. The HXB2 subtype B prototype strain was used as outgroup. In order to study potential bias due to drug-induced convergent evolution, all our analysis were also repeated on an alignment for which we removed 43 codon positions known to be associated with major resistance mutations according to the WHO-list of 2009 [45]. The following positions were excluded for protease (23, 24, 30, 32, 46, 47, 48, 50, 53, 54, 73, 76, 82, 83, 84, 85, 88, 90) and RT (41, 65, 67, 69, 70, 74, 75, 77, 100, 101, 103, 106, 115, 116, 151, 179, 181, 184, 188, 190, 210, 215, 219, 225, 230), leaving 882 nt in the final alignment. Both complete (1,011 nt) and restricted (882 nt) sequence alignments are available from the authors upon request. Maximum Likelihood phylogenies were inferred using the GTR+I+Γ4 nucleotide substitution model recommended by [46] and implemented in PhyML v3.0 [47]. The SPR option was selected to search the tree space and aLRT SH-like branch supports were used to assess confidence in topology [48]. The phylogenetic tree was drawn with FIGTREE (tree.bio.ed.ac.uk/software/figtree/).

In order to better determine and visualize the relationship of the subtype C sequences from Senegal to those from other geographic areas, another phylogenetic analysis was performed with less sequences. For this subtree, we collected from the large, previous phylogenetic tree, all descendant sequences of nodes that are first or second level ancestor of at least one sequence from Senegal (i.e., all Senegalese sequences plus their sisters and close relatives). A phylogeny was then inferred, using the same method and options as described above, but in addition to aLRT we ran a non-parametric bootstrap with 100 replicates to obtain a second assessment of branch supports. A phylogenetic analysis on this subset of sequences was also inferred using MrBayes v3.1 [49] with the same substitution model as for the maximum likelihood tree, and with chain length and tree sampling frequency of 5×10^7^ and 1×10^4^ generations, respectively. A burn-in of 2,000 sampled trees (i.e. ∼40%) was selected. By the end of the run, the average standard deviation of split frequencies was below 0.01 and the potential scale reduction factor of every parameter was in the range [0.999, 1.001], except the parameter pinvar which is at 1.002, proving the convergence of the Markov chains (see MrBayes manual).

### Dating the introduction of subtype C in Senegal and MSM population

Estimates of the substitution rate and dates of the most recent common ancestor (MRCA) of subtype C in Senegal and in the sub-epidemic in MSM were obtained using BEAST v1.6.1 [50]. The 56 *pol* gene subtype C sequences from Senegal were analyzed under a GTR+I+Γ4 substitution process (as for phylogenetic analyzes). We used three different molecular clock models (strict clock, relaxed uncorrelated exponential and relaxed uncorrelated lognormal) [51] as implemented in BEAST with a Bayesian skyride tree prior as a coalescent demographic model with time-aware smoothing [52]. For the parameters of each molecular clock model (ucld.mean, uced.mean and clock.rate for the relaxed lognormal, relaxed exponential and strict molecular clock respectively) we tested a total of four different priors, one non-informative prior based on a uniform distribution (between 0.0 and 1.0) and three priors with varying information levels based on normal distribution with a mean of 2.5×10^−3^ (based on estimations from a previous study [27] in the same genomic region and as estimated by Path-O-Gen: tree.bio.ed.ac.uk/software/pathogen/) and standard deviations of 10×10^−4^, 7.5×10^−4^, and 5.0×10^−4^, respectively. For the ucld.stdev parameter (representing the variability of the rates among branches for the relaxed lognormal molecular clock) we used a prior based on an exponential distribution with mean of 0.1 (personal communication with A. Drummond). MCMC simulations were run for 2.5×10^8^ chain steps with sub-sampling every 2.5×10^5^ steps. Convergence of the chains was inspected using Tracer v.1.5. For each tested prior and for each parameter, effective sample size (ESS) values were always above 300. The Bayes Factor was calculated to compare molecular clock models, using marginal likelihood as implemented in Tracer v.1.5. The Maximum Clade Credibility with time scale (MCC) tree was obtained by TreeAnnotator v1.6.1 with a burn-in of the first hundred trees.

## Supporting Information

Figure S1 **Maximum likelihood phylogenetic tree based on 3,081 HIV-1 subtype C** ***pol*** **sequences, without codons associated to drug resistance in PR and RT.** Maximum likelihood phylogenetic tree (PhyML, with the same options as for the tree in Figure 1) based on 882 nucleotide sites of *pol* gene sequence from 3,081 HIV-1 subtype C isolates; nucleotide sites with coordinates 2,253–3,263 of HXB2 are included, but codon positions known to be associated with major resistance mutations according to the WHO-list of 2009 were removed (see Materials and Methods). Sequences were isolated in the countries shown in Table 2. Sequences are colored according to their region of origin: Senegal in red, Southern African countries (South-Africa, Botswana, Malawi, Mozambique, Swaziland, Zambia and Zimbabwe) in orange, other African countries (mainly from the East) in yellow, North and South America in purple, Asia in green and Europe in blue. The branch support (aLRT) of clades A, B, C and MSM are respectively of 94%, 92%, 83% and 96%.
(JPG)

Figure S2 **Maximum likelihood phylogenetic tree constructed of 56 HIV-1 C** ***pol*** **sequences from Senegal and 121 close relatives.** Detailed maximum likelihood (PhyML) phylogenetic tree constructed using 1,011 nucleotide sites of *pol* gene sequence (nucleotides 2,253–3,263 of HXB2 coordinates) from 177 HIV-1 subtype C isolates from Senegal and close relatives (see Materials and Methods) as shown in Figure 2 but names of the strains are added. Branch support values (bootstrap and aLRT) are displayed (see figure legend). Colors indicate the geographic origin and sequences were isolated in the following countries: 56 in red from Senegal, 25 in orange from Zambia, 49 in yellow from southern Africa (Botswana 6; Mozambique 5; Swaziland 2; South Africa 35; Zimbabwe 1), 12 in green from East Africa (Burundi 2; Ethiopia 9; Kenya 1; Sudan 2), 3 in blue from other African countries (DRC 1; Equatorial Guinea 1; Gabon 1) and 30 in black from European and Asian countries (Belgium 4; China 1; Germany 2; Denmark 1; Spain 5; France 1; Greece 1; Israel 1; India 1; Italia 1; Luxembourg 1; Norway 2; Portugal 2; Sweden 7).
(TIFF)

Figure S3 **Bayesian phylogenetic tree of 56 HIV-1 C** ***pol*** **sequences from Senegal and 121 close relatives.** Detailed Bayesian phylogenetic tree (MrBayes, same model and similar options as for the tree in Figure 2, see Materials and Methods) constructed using 1,011 nucleotide sites of *pol* gene sequence (nucleotides 2,253–3,263 of HXB2 coordinates) from 177 HIV-1 subtype C isolates from Senegal and close relatives. Clades with posterior probabilities ≥95% are shown. Colors indicate the geographic origin of the sequences, which were isolated in the following countries: 56 in red from Senegal, 25 in orange from Zambia, 49 in yellow from southern Africa (Botswana 6; Mozambique 5; Swaziland 2; South Africa 35; Zimbabwe 1), 12 in green from East Africa (Burundi 2; Ethiopia 9; Kenya 1; Sudan 2), 3 in blue from other African countries (DRC 1; Equatorial Guinea 1; Gabon 1) and 30 in black from European and Asian countries (Belgium 4; China 1; Germany 2; Denmark 1; Spain 5; France 1; Greece 1; Israel 1; India 1; Italia 1; Luxembourg 1; Norway 2; Portugal 2; Sweden 7).
(TIFF)

Figure S4 **Bayesian tree with timescale of 56 HIV-1 C** ***pol*** **sequences from Senegal, without sites associated to major, known resistance in PR and RT.** Maximum clade credibility tree with time scale obtained with BEAST using 1,011 nucleotide sites of *pol* gene sequences (nucleotides 2,253–3,263 of HXB2 coordinates) from 56 HIV-1 subtype C isolates from Senegal. This tree is obtained using the relaxed uncorrelated lognormal molecular clock model and moderately informative substitution rate prior (Normal: 2.5×10^−3^, 7.5×10^−4^). Clades with posterior probabilities ≥95% are indicated by diamonds. MSM isolates are colored in red.
(TIFF)

Table S1 **Genbank accession numbers per country of subtype C HIV-1 strains included in the study.**
(DOC)

Table S2 **Details of the strains included in the restricted phylogenetic tree analysis from** **Figures 2** **, S2 and S3.**
(PDF)

Table S3 **Dating the subtype C epidemic in general and MSM populations in Senegal.** Coalescent based estimations (BEAST) and 95% highest posterior density (HPD) intervals of the MRCA dates and substitution rates of 56 HIV-1 subtype C *pol* sequences obtained from the general and the MSM population. Results are displayed for all tested substitution rate priors and molecular clock models.
(PDF)
