# Supplementary figures and images for: The Origin and Evolutionary History of HIV-1 Subtype C in Senegal
Source: PLoS One. 2012 Mar 28;7(3):e33579. doi: 10.1371/journal.pone.0033579 (PMC3314668; doi:10.1371/journal.pone.0033579)

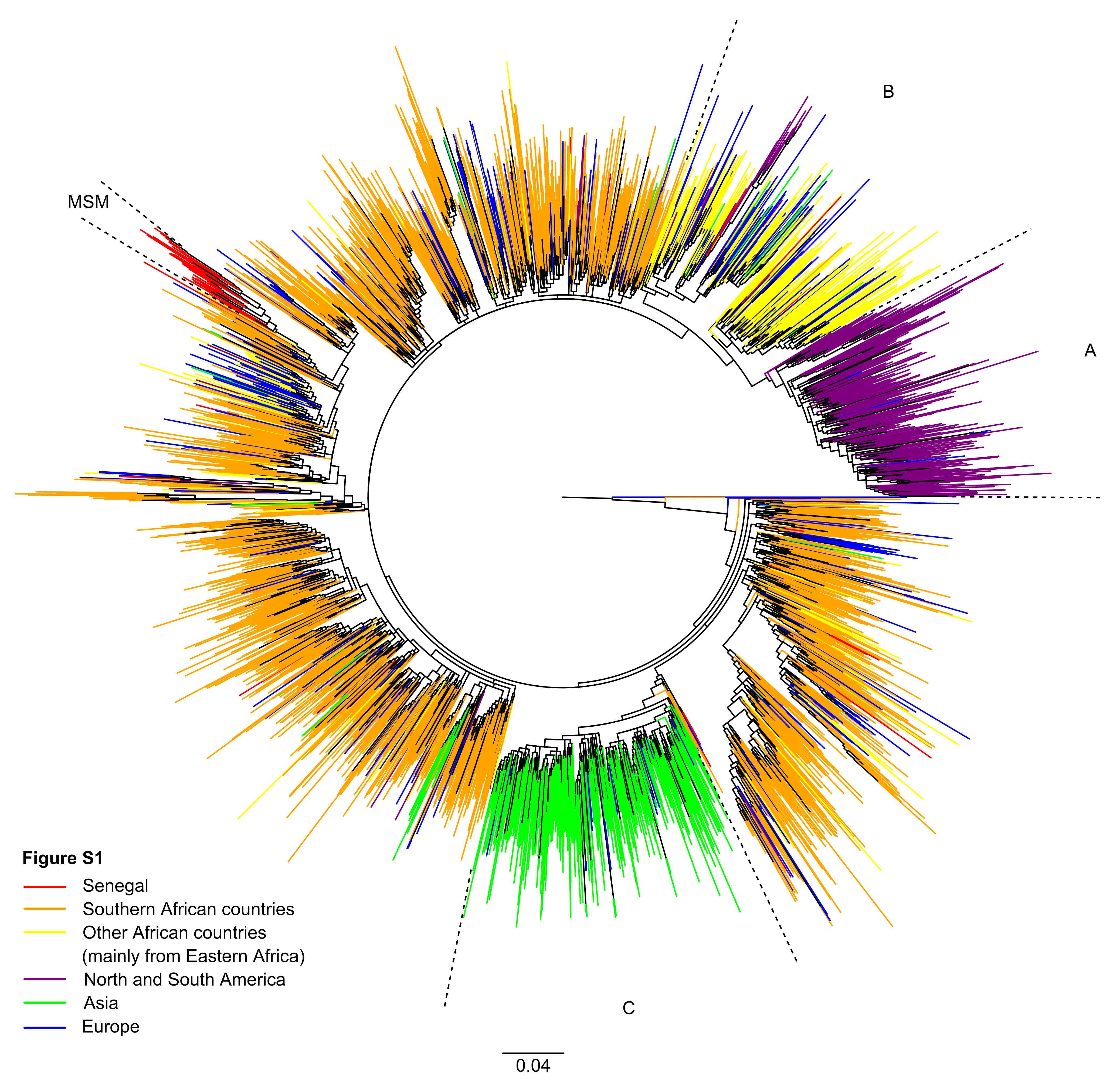

Supplement: Figure S1 — Maximum likelihood phylogenetic tree based on 3,081 HIV-1 subtype C pol sequences, without codons associated to drug resistance in PR and RT. Maximum likelihood phylogenetic tree (PhyML, with the same options as for the tree in Figure 1) based on 882 nucleotide sites of pol gene sequence from 3,081 HIV-1 subtype C isolates; nucleotide sites with coordinates 2,253–3,263 of HXB2 are included, but codon positions known to be associated with major resistance mutations according to the WHO-list of 2009 were removed (see Materials and Methods). Sequences were isolated in the countries shown in Table 2. Sequences are colored according to their region of origin: Senegal in red, Southern African countries (South-Africa, Botswana, Malawi, Mozambique, Swaziland, Zambia and Zimbabwe) in orange, other African countries (mainly from the East) in yellow, North and South America in purple, Asia in green and Europe in blue. The branch support (aLRT) of clades A, B, C and MSM are respectively of 94%, 92%, 83% and 96%. (JPG) [file pone.0033579.s001.jpg]

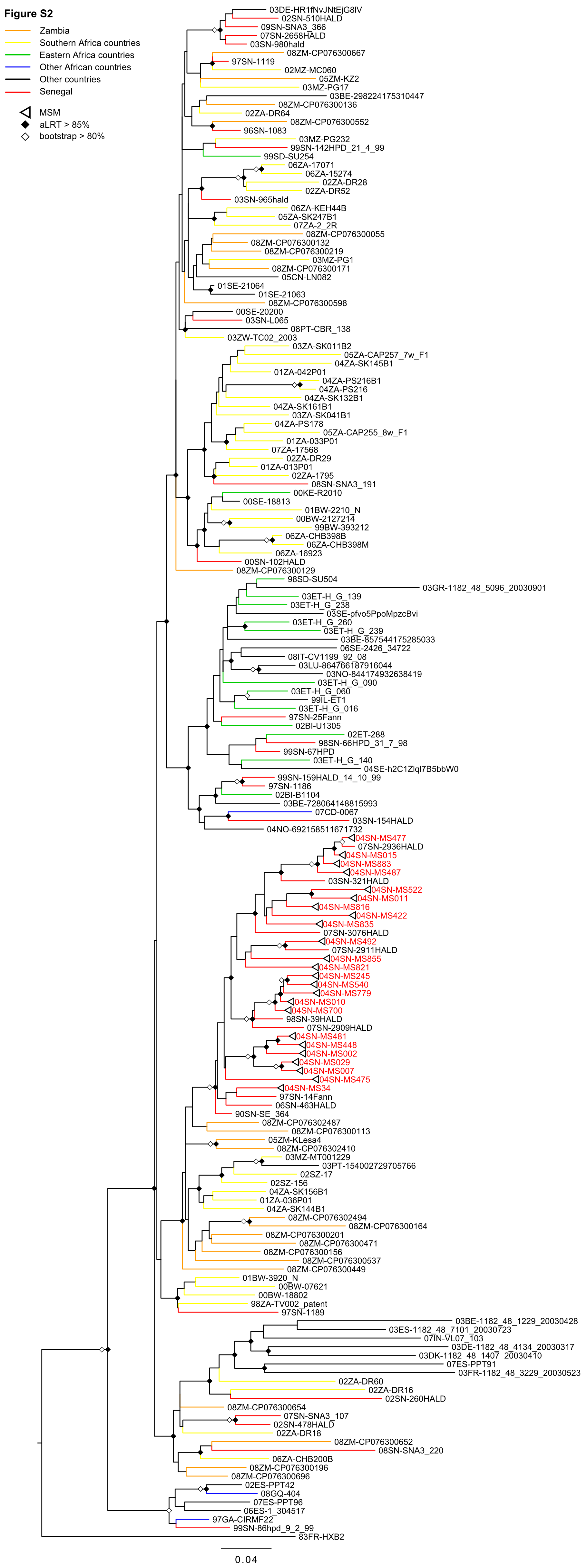

Supplement: Figure S2 — Maximum likelihood phylogenetic tree constructed of 56 HIV-1 C pol sequences from Senegal and 121 close relatives. Detailed maximum likelihood (PhyML) phylogenetic tree constructed using 1,011 nucleotide sites of pol gene sequence (nucleotides 2,253–3,263 of HXB2 coordinates) from 177 HIV-1 subtype C isolates from Senegal and close relatives (see Materials and Methods) as shown in Figure 2 but names of the strains are added. Branch support values (bootstrap and aLRT) are displayed (see figure legend). Colors indicate the geographic origin and sequences were isolated in the following countries: 56 in red from Senegal, 25 in orange from Zambia, 49 in yellow from southern Africa (Botswana 6; Mozambique 5; Swaziland 2; South Africa 35; Zimbabwe 1), 12 in green from East Africa (Burundi 2; Ethiopia 9; Kenya 1; Sudan 2), 3 in blue from other African countries (DRC 1; Equatorial Guinea 1; Gabon 1) and 30 in black from European and Asian countries (Belgium 4; China 1; Germany 2; Denmark 1; Spain 5; France 1; Greece 1; Israel 1; India 1; Italia 1; Luxembourg 1; Norway 2; Portugal 2; Sweden 7). (TIFF) [file pone.0033579.s002.tif]

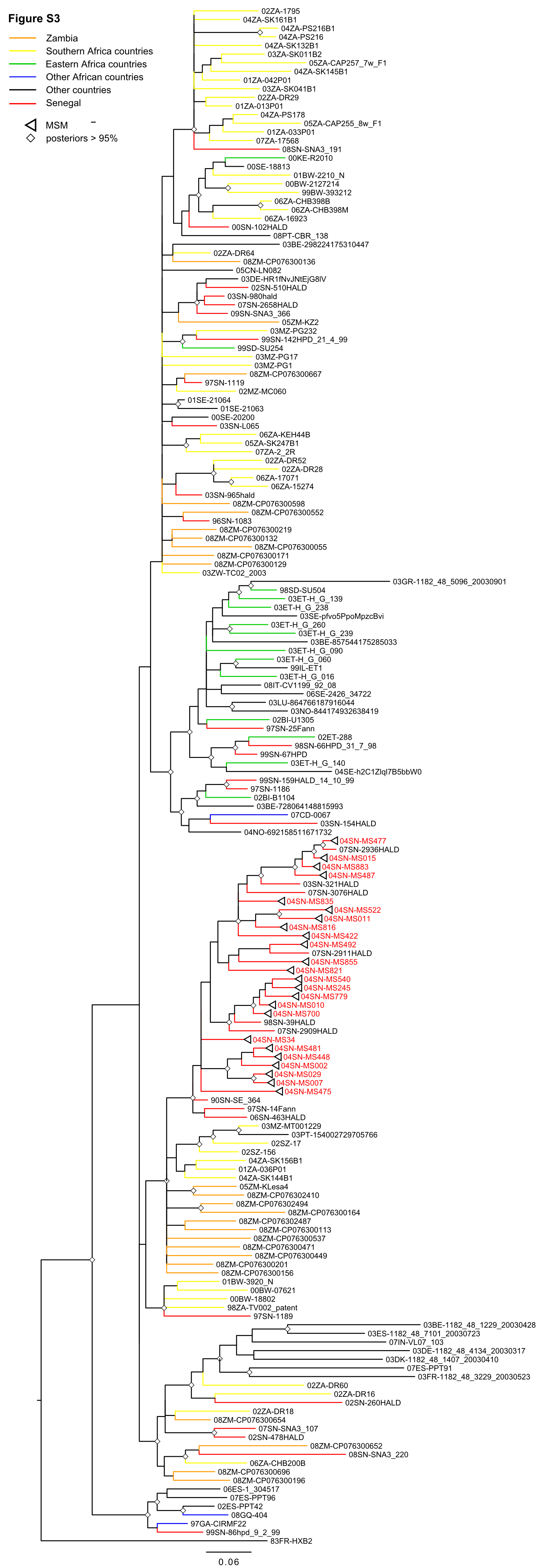

Supplement: Figure S3 — Bayesian phylogenetic tree of 56 HIV-1 C pol sequences from Senegal and 121 close relatives. Detailed Bayesian phylogenetic tree (MrBayes, same model and similar options as for the tree in Figure 2, see Materials and Methods) constructed using 1,011 nucleotide sites of pol gene sequence (nucleotides 2,253–3,263 of HXB2 coordinates) from 177 HIV-1 subtype C isolates from Senegal and close relatives. Clades with posterior probabilities ≥95% are shown. Colors indicate the geographic origin of the sequences, which were isolated in the following countries: 56 in red from Senegal, 25 in orange from Zambia, 49 in yellow from southern Africa (Botswana 6; Mozambique 5; Swaziland 2; South Africa 35; Zimbabwe 1), 12 in green from East Africa (Burundi 2; Ethiopia 9; Kenya 1; Sudan 2), 3 in blue from other African countries (DRC 1; Equatorial Guinea 1; Gabon 1) and 30 in black from European and Asian countries (Belgium 4; China 1; Germany 2; Denmark 1; Spain 5; France 1; Greece 1; Israel 1; India 1; Italia 1; Luxembourg 1; Norway 2; Portugal 2; Sweden 7). (TIFF) [file pone.0033579.s003.tif]

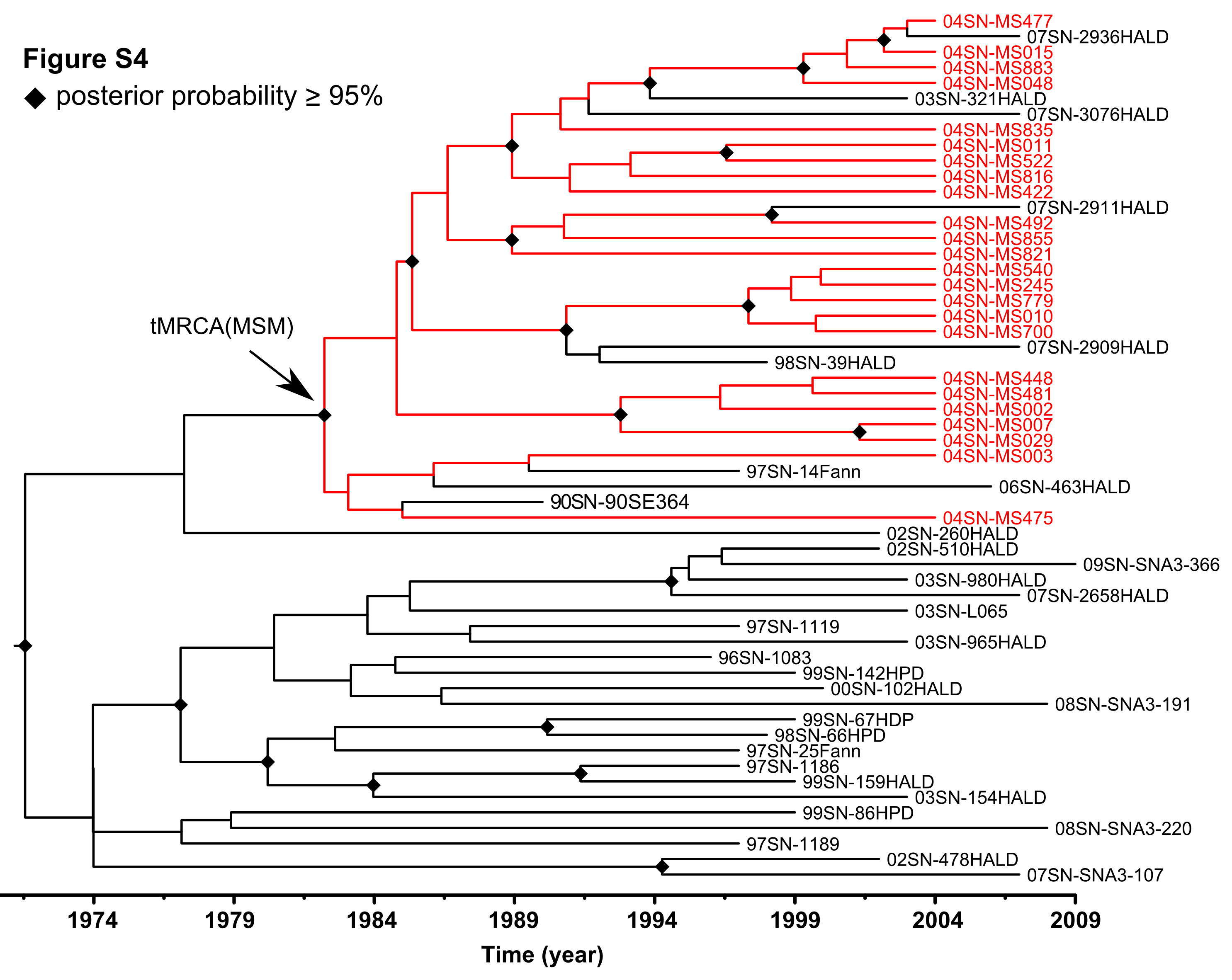

Supplement: Figure S4 — Bayesian tree with timescale of 56 HIV-1 C pol sequences from Senegal, without sites associated to major, known resistance in PR and RT. Maximum clade credibility tree with time scale obtained with BEAST using 1,011 nucleotide sites of pol gene sequences (nucleotides 2,253–3,263 of HXB2 coordinates) from 56 HIV-1 subtype C isolates from Senegal. This tree is obtained using the relaxed uncorrelated lognormal molecular clock model and moderately informative substitution rate prior (Normal: 2.5×10^−3^, 7.5×10^−4^). Clades with posterior probabilities ≥95% are indicated by diamonds. MSM isolates are colored in red. (TIFF) [file pone.0033579.s004.tif]
