# Supplementary material for: The Origin and Evolutionary History of HIV-1 Subtype C in Senegal
Source: PLoS One. 2012 Mar 28;7(3):e33579. doi: 10.1371/journal.pone.0033579 (PMC3314668; doi:10.1371/journal.pone.0033579)
Supplement: Table S1 — Genbank accession numbers per country of subtype C HIV-1 strains included in the study. (DOC) [file pone.0033579.s005.doc]

**TABLE S1 : Genbank accession numbers per cpuntry of subtype C HIV-1 strains included in the study.**

**Argentina**

EF120337, EF120336, EF120254, EF120244

**Austria**

GQ400369, GQ400143, GQ399796

**Belgium**

AF338992, AF338990, AF338984, DQ877762, AY749181, AY749180, AY749179, AY749177, AY749174, AY749172, AY749171, AY749170, AY749169, EU248490, EU248485, EU248453, EU248441, EU248397, EU248393, EU248376, EU248356, EU248348, EU248333, EU248298, EU248293, EU248586, EU248584, EU248569, EU248558, EU248545, EU248543, EU248524, EU248523, EU248516, EU248493

**Botswana**

AF290030, AF290027, AY829338, AY829337, AY829336, AY829335, AY829334, AY829333, AY829332, AY829331, AY829330, AY829329, AY829328, AY829327, AY829326, AY829325, AY829324, AY829323, AY829322, AY829321, AY829320, AY829319, AY829318, AY829317, AY829316, AY829315, AY829314, AY829313, AY829312, AY829311, AY829310, AY829309, AY829308, AY829307, AY829306, AY829305, AY829304, AY829303, AY829302, AY829301, AY829300, AY829299, AY829298, AY829297, AY829296, AY829295, AY829294, AY829293, AY829292, AY829291, AY829290, AY829288, AY829287, AY829286, AY829285, AY829284, AY829283, AY829282, AY829281, AY829280, AY829279, AY829278, AY829277, AY829276, AY829275, AY829274, AY829273, AY829272, AY829271, AY829270, AY829269, AY829268, AF443115, AF443114, AF443113, AF443112, AF443111, AF443110, AF443109, AF443108, AF443107, AF443105, AF443104, AF443103, AF443102, AF443101, AF443100, AF443099, AF443098, AF443097, AF443096, AF443095, AF443094, AF443093, AF443092, AF443091, AF443090, AF443089, AF443088, AF443087, AF443086, AF443085, AF443084, AF443083, AF443082, AF443081, AF443080, AF443079, AF443078, AF443077, AF443076, AF443075, AF443074, AF321523, AF110981, AF110980, AF110978, AF110977, AF110976, AF110974, AF110972, AF110971, AF110970, AF110969, AF110968, AF110967, AF110966, AF110965, AF110964, AF110963, AF110962, AF110961, AF110959

**Brazil**

GQ294621, HQ127603, HQ127596, HQ127560, HQ127550, HQ127529, HQ127526, HQ127522, HQ127521, HQ127515, HQ127499, HQ127462, EU340754, EU340751, EU340749, EU340747, EU340746, EU340744, EU340738, EU340736, EU340730, EU340725, EU340722, EU340715, EU340713, EU340711, EU340709, EU340708, FJ591976, FJ591975, FJ591967, FJ591958, FJ591957, FJ591955, FJ591954, FJ591953, FJ591920, FJ591899, FJ591853, FJ591836, FJ591731, FJ591716, FJ591651, FJ591648, FJ591606, FJ591605, FJ591602, FJ591597, FJ591594, FJ591592, FJ591590, FJ591578, FJ591574, FJ591563, FJ591561, FJ591560, FJ591557, FJ591555, FJ591552, FJ591551, FJ591548, FJ591545, FJ591541, FJ591531, FJ591529, FJ591527, FJ591526, FJ591524, FJ591520, FJ591507, FJ591506, FJ591489, FJ591475, FJ591470, FJ591468, FJ591466, FJ591458, FJ591456, FJ591455, FJ591444, FJ591441, FJ591440, FJ591436, FJ591430, FJ591425, FJ591423, FJ591420, FJ591417, FJ591414, FJ591413, FJ591411, FJ591403, FJ591402, FJ591393, FJ591392, FJ591389, FJ591388, FJ591387, FJ591381, FJ591377, FJ591376, FJ591374, FJ591368, FJ591344, FJ591342, FJ591339, FJ591334, FJ591331, FJ591327, FJ591324, FJ591320, FJ591318, FJ591317, FJ591316, FJ591315, FJ591306, FJ591303, FJ591302, FJ591296, FJ591293, FJ591292, FJ591290, FJ591289, FJ591287, FJ591281, FJ591279, FJ591277, FJ591268, FJ591264, FJ591260, FJ591255, FJ591248, FJ591242, FJ591238, FJ591232, FJ591225, FJ591219, FJ591217, FJ591215, FJ591214, FJ591212, FJ591210, FJ591203, FJ591196, FJ591195, FJ591190, FJ591188, FJ591181, FJ591180, FJ591176, U52953, FJ784207, FJ784199, AY390190, AY390188, FJ548791, HM025482, HM025475, HM024864, HM534197, HM534190, HM534185, HM534176, HM534131, HM534122, HM534117, HM534088, HM534043, HM534030, HM534022, HM534020, HM534017, GQ401330, GQ401326, GQ401324, GQ401322, GQ401321, GQ401320, GQ401315, GQ401312, GQ401311, GQ401309, GQ401308, GQ401305, GQ401304, GQ401303, GQ401302, GQ401301, GQ401300, GQ401299, GQ862311, EF379213, EF379212, EF379211, EF379209, EF379207, EF379206, EF379204, EF379203, EF379199, EF379198, EF379195, EF379193, EF379191, EF379181, EF379178, EF379177, EF379176, EF379174, EF379171, EF379168, EF379159, EF379154, AY727525, AY727524, AY727523, AY727522, DQ518545, DQ518543, DQ518540, DQ518539, DQ518538, DQ518537, DQ518536, DQ518533, DQ518532, DQ518531, DQ518527, DQ518524, DQ518523, DQ518520, DQ518486, DQ518484, DQ518479, AF286228, EU293534, DQ191009, DQ191006, DQ191005, DQ190959, DQ190951, DQ344011, DQ344006, DQ344005, DQ344003, DQ344002, DQ344001, DQ343999, DQ343986, AY213550, AY213542, AY213522, AY213521

**Burundi**

AM260284, AM260265, AM260236, AM260321, AM260320, AM260318, AM260317, AM260316, AM260314, AM260312, AM260311, AM260310, AM260309, AM260308, AM260307, AM260306, AM260305, AM260304, AM260303, AM260302, AM260301, AM260300, AM260299, AM260298, AM260297, AM260296, AM260295, AM260294, AM260293, AM260292, AM260291, AM260290, AM260288, AM260287, AM260286, AM260285, AM260283, AM260282, AM260281, AM260280, AM260279, AM260278, AM260277, AM260276, AM260275, AM260274, AM260273, AM260272, AM260271, AM260270, AM260269, AM260268, AM260267, AM260266, AM260264, AM260263, AM260262, AM260261, AM260259, AM260258, AM260257, AM260256, AM260252, AM260251, AM260250, AM260249, AM260248, AM260247, AM260246, AM260245, AM260244, AM260243, AM260242, AM260241, AM260239, AM260238, AM260237, AM260235, AM260234, AM260233, AM260232, AM260229, AM260228, AM260227, AM260226, AM260225, AM260223, AM260222, AM260221, AM260220, AM260219

**China**

EF122539, FJ531453, FM251950, FM251949, FJ387048, FJ387034, AY967806

**Cuba**

HQ108352, DQ113384, DQ113379, DQ113364, DQ113348, DQ113317, DQ113298, DQ113243, DQ113224, DQ113190, DQ113165, DQ113161, DQ113130, DQ113129, DQ113111, DQ113094, DQ113084, DQ113072, DQ113042, DQ113033, DQ113013, DQ112999, DQ112986, DQ112982, DQ112972

**Cyprus**

FJ388952, FJ388948, FJ388913, FJ388901, EU673407, EU673406, EU673391, EU673374

**Czech Republic**

AY694340, AY694304, AY694300, AY694299, AY694298, AY694280, AY694266, AY694256, AY694255, AY694240, AY694221

**Democratic Republic of Congo**

AM041050, AM041034, AM041017, AM041011, AM041006, AM041002, AM040991, AM040985, FR666658, FR666650, FR666649, FR666642, FR666631, FR666625, FR666621, FR666620, FR666617, FR666613, FR666608

**Denmark**

AM285286, AM933272, DQ877827, DQ877803, GQ400527, GQ400526, GQ400501, GQ400394, GQ400288, GQ400044, GQ399711, GQ399697, GQ399616, GQ399556, GQ399517, GQ399252, GQ399228, GQ399200, GQ399028, GQ398909, EF514713

**Djibouti**

AF447839

**Equatorial Guinea**

FN557338

**Eritrea**

AY371692, AY371691

**Ethiopia**

AB285843, AB285842, AB285841, AB285840, AB285839, AB285838, AB285837, AB285836, AB285835, AB285834, AB285833, AB285832, AB285829, AB285828, AB285827, AB285826, AB285825, AB285824, AB285823, AB285822, AB285821, AB285820, AB285819, AB285818, AB285817, AB285816, AB285815, AB285814, AB285813, AB285812, AB285811, AB285810, AB285809, AB285808, AB285807, AB285806, AB285805, AB285804, AB285803, AB285802, AB285801, AB285800, AB285799, AB285798, AB285797, AB285796, AB285795, AB285794, AB285793, AB285792, AB285791, AB285790, AB285789, AB285788, AB285786, AB285784, AB285783, AB285782, AB285781, AB285780, AB285779, AB285778, AB285777, AB285775, AB285774, AB285773, AB285772, AB285771, AB285770, AB285769, AB285768, AB285767, AB285766, AB285765, AB285764, AB285763, AB285762, AB285761, AB285760, AB285759, AB285758, AB285757, AB285756, AB285755, AB285754, AB285753, AB285752, AY713417, AY371693, AF447841, AY242598, AY242592, AY242589, AY242586, AY242582, AY242581, AY242591, AY242594, U46016

**Finland**

GQ400095, GQ400025, GQ399414, GQ399408, GQ399298, GQ398837

**France**

AJ270550, AJ578177, AJ577965, AJ577964, AJ577870, AJ287041, DQ878136

**Gabon**

AY140617

**Georgia**

DQ207941

**Germany**

GU271173, GU271172, DQ878365, DQ878240, GQ400682, GQ400661, GQ400542

**Greece**

DQ878592, DQ878534, GQ399518

**Honduras**

EU312784

**India**

EU744914, EU744913, FJ817442, FJ817435, GQ906416, GQ906409, FJ907488, FJ907469, FJ907466, GQ891861, HQ456682, HQ456680, HQ456679, HQ456678, HQ456676, HQ456675, HQ456674, HQ456673, HQ456672, HQ456671, HQ456670, HQ456669, HQ456668, HQ456667, AY049711, AY049709, AY049708, AB023804, EF469243, AY713414, AF067159, AF067158, AF067157, AF067155, AF067154, FJ878970, FJ878969, FJ878968, FJ878967, FJ878966, FJ878965, FJ878964, FJ878963, FJ878962, FJ878961, FJ878960, FJ878959, FJ878958, FJ878957, FJ878956, FJ878955, FJ878953, FJ878952, FJ878951, FJ878950, FJ878949, FJ878948, FJ878947, FJ878946, FJ878945, FJ878944, FJ878943, FJ878942, FJ878941, FJ878940, FJ878939, FJ878938, FJ878937, EU683801, EU683800, EU683799, EU683798, EU683797, EU683796, EU683795, EU683794, EU683793, EU683792, EU683791, EU683790, EU683789, EU683788, EU683787, EU683786, EU683785, EU683784, EU683783, EU683782, EU683781, EU683780, EU683779, EU683778, EU683777, EU683776, EU683775, EU683774, EU683773, EU683772, EU683771, EU683770, EU683769, EU683768, EU683767, EU683766, EU683765, EU683764, EU683763, EU683762, EU683761, EU683759, EU683758, EU683757, EU683756, EU683755, EU683754, EU683753, EU683752, EU744912, EU110083, EU110081, EU110080, EU110079, EU110078, EU110077, EU110076, EU110075, EU110074, EU110073, EU110072, EU110071, EU110070, EU110069, EU110068, EU110067, EU110066, EU110065, EU110064, EU110063, EU110062, EU110061, EU110060, EU110059, EU110058, EU110057, EU110056, EU110055, EU110054, EU110053, EU106126, EU106125, EU106124, EU106123, EU106122, EU106121, EU106120, EU106119, EU106118, EU106117, EU037779, EU037774, EU037773, EU037778, EU037776, EU030418, EU030417, EU030416, EU030415, EU030414, EU030413, EU030412, EU030411, EU030410, EU781846, EU781845, EU781844, EU781843, EU781842, EU781841, EU781840, EU781839, EU781838, EU781837, EU781836, EU781835, EU781834, EU158892, EU158891, EU158890, EU158889, EU158888, EU158886, EU158885, EU158882, EU158881, EU158880, EU158879, EU158878, EU158877, EU158876, EU158875, EU158874, EU158873, EU158872, EU158871, EU158870, EU158869, EU158868, EU158867, EU158866, EU158865, EU484324, EU484323, EU484322, EU447781, EU447780, EU447779, EU447778, EU447776, DQ826621, DQ826629, DQ826668, DQ826667, DQ826665, DQ826663, DQ826662, DQ826660, DQ826659, DQ826658, DQ826657, DQ826656, DQ826655, DQ826653, DQ826652, DQ826651, DQ826649, DQ826647, DQ826646, DQ826645, DQ826644, DQ826643, DQ826642, DQ826641, DQ826640, DQ826639, DQ826638, DQ826637, DQ826636, DQ826635, DQ826633, DQ826632, DQ826631, DQ826630, DQ826628, DQ826627, DQ826626, DQ826625, DQ826624, DQ826623, DQ826622, DQ826620, DQ826619, DQ826617, DQ826616, DQ826615, DQ826614, DQ826613, DQ826612, DQ826611, DQ826609, DQ826607, DQ826606, DQ826605, DQ826603, DQ826602, DQ826601, DQ826600, DQ826599, DQ826598, DQ826597, DQ826596, EF186988, EF186987, EF186986, EF186985, EF186984, EF186983, EF186982, EF186980, EF186979, EF186978, EF186977, EF186976, EF186975, EF186974, EF186971, EF186970, EF186969, EF186968, EF186967, EF186966, EF186965, EF186964, EF186963, EF186962, EF186960, EF186957, EF186956, EF186955, EF186954, EF186953, EF186952, EF186951, EF186950, EF186949, EF186948, EF186947, EF186946, EF186944, EF186943, EF186942, EF186941, EF186940, EF186939, EF186938, EF186936, EF186935, EF186934, EF186933, EF186932, EF186930, EF186928, EF186927, EF186926, AY787536, AY787535, AY787532, AY787522, AY787492, AY746386, AY746385, AY746384, AY746381, AY746380, AY746377, AY746374, AY746373, AY746368, AY746367, AY746363, AY746361, AY739913, AY739911, AF286232, AF286231, AF286223

**Israel**

AY255826, AY255825, AY255824, AY255823, AF286233

**Italy**

AY372167, AY372166, AY372165, AY460120, AY377432, AY377430, AY359514, AY359513, GQ399666, GQ399122, GQ398966, FJ030696, AF295284, GU969571, GU969550, GU969545, GU969543, GU969541, GU969538, GU969533, GU969524, GU969505

**Kenya**

HM164129, AY945738, AY492764, AF457054

**Luxemburg**

GQ400273, GQ399890, GQ399768

**Malawi**

AY713413, AY756889, AY756888, AY756887, AY756886, AY756885, AY756884, AY756882, AY756878, AY756877, AY756876, AY756874, AY756873, AY756872, AY756871, AY756870, AY756869, AY756867, AY756865, AY756864, AY756863, AY756862, AY756860, AY756859, AY756858, AY756854, AY756853, AY756851, AY756850, AY756849, AY756847, AY756844, AY756841, AY756840, AY756839, AY756836, AY756834, AY756833, AY756831, AY756830, AY756827, AY756826, AY756825, AY756823, EF602625, EF602623, EF602622, EF602621, EF602620, EF602616, EF602614, EF602611, EF602610, EF602607, EF602606, EF602605, EF602604, EF602601, EF602599, EF602598, EF602596, EF602595, EF602592, EF602591, EF602590, EF602589, EF602588, EF602586, EF602585, EF602584, EF602583

**Mali**

EF064480

**Mozambique**

EU856363, GU199582, GU199581, GU199580, GU199579, GU199578, GU199577, GU199576, GU199575, GU199574, GU199573, GU199572, GU199571, GU199570, GU199569, GU199568, GU199567, GU199566, GU199565, GU199564, GU199563, GU199562, GU199561, GU199560, GU199559, GU199558, GU199557, GU199556, GU199555, GU199554, GU199553, GU199552, GU199551, GU199549, GU199548, GU199547, GU199545, GU199544, GU199543, GU199542, GU199541, GU199540, GU199539, GU199538, GU199537, GU199536, GU199535, GU199534, GU199533, GU199532, GU199531, GU199530, GU199529, GU199528, GU199527, GU199526, AM071454, AM071453, AM071452, AM071451, AM071450, AM071449, AM071448, AM071447, AM071446, AM071445, AM071444, AM071443, AM071442, AM071441, AM071440, AM071439, AM071437, AM071436, AM071435, AM071434, AM071433, AM071432, AM071431, AM071430, AM071429, AM071428, AM071427, AM071426, AM071425, AM071424, AM071423, AM071422, AM071421, AM071420, AM071419, AM071418, AM071417, AM071416, AM071415, AM071414, AM071413, AM071412

**Myanmar**

AB097871

**Niger**

HQ843636, HQ843518, DQ013277, DQ013271

**Norway**

GQ400496, GQ400431, GQ400428, GQ400400, GQ400339, GQ400237, GQ400151, GQ399985, GQ399970, GQ399875, GQ399733, GQ399501, GQ399394, GQ399345, GQ399127, GQ398881

**Uganda**

DQ079829, GQ409622, GQ409590, GQ409573, AF388154, AF388161, AF388136, AF388102, AY435369, AY435293, AY435285, HM037812, EU306757, AF447844, FJ389056, GU059310

**Philippines**

AB587103

**Poland**

GU906870, GU906868

**Portugal**

HM102339, HM102338, HM102337, HM102336, HM102335, HM102334, HM102333, HM102332, HM102331, HM102330, GQ400298, GQ400211, GQ400193, GQ400145, GQ400064, GQ399972, GQ399932, GQ399801, GQ399593, GQ399507, GQ399364, GQ399346, GQ399069, FJ360883, FJ360882, FJ360881, FJ360879, FJ360877

**Romania**

HM191557, HM191556, HM191555, HM191554, HM191553, HM191552, HM191551, HM191550, HM191549, HM191548, HM191547, HM191546, HM191545, HM191544, HM191543, HM191542, HM191541, HM191540, HM191539, HM191538, HM191537, HM191536, HM191535, HM191534, HM191533, HM191532, HM191531, HM191530, HM191529, HM191528, HM191527, HM191526, HM191525, HM191524, HM191522

**Russia**

EU345720

**Senegal**

HM002544, HM002517, HM002515, HM002507, FM210753, FM210752, FM210749, FM210745, FM210741, FM210740, FM210737, FM210736, FM210726, FM210725, FM210723, FM210722, FM210718, FM210717, FM210716, FM210712, FM210709, FM210699, FM210691, FM210689, FM210687, FM210686, FM210685, FM210684, FN599776, FN599773, FN599737, FN599718, AJ583722, AJ583716, AJ583715, AJ583739, AJ287005, AY713416, HE588158, HE588157, HE588156, HE588149, HE588155, HE588150, HE588151, HE588152, HE588153, HE588154, HE588159, HE588162, HE588163, HE588165, HE588164, HE588166, HE588161, HE588160

**Slovakia**

GQ399752

**Somalia**

AF447850

**Sudan**

AY102510, AY102507, AY102506, AY102505, AY102502, AY102498, AY102497, AY102491, AY102489, AY102486

**South Africa**

AY158535, AY158534, AY158533, GQ999991, GQ999990, GQ999989, GQ999988, GQ999987, GQ999986, GQ999985, GQ999984, GQ999983, GQ999982, GQ999981, GQ999980, GQ999979, GQ999978, GQ999977, GQ999976, GQ999975, GQ999974, GQ999973, GQ999972, AY136956, AY136955, AY136954, EU854561, EU854559, EU854553, EU854550, EU854548, EU854545, EU854543, EU854540, EU854539, EU854538, EU854536, EU854532, EU854529, EU854528, EU854526, EU854525, EU854524, EU854522, EU854520, EU854518, EU854517, EU854513, EU854509, EU854507, EU854503, EU854501, EU854498, EU854497, EU854495, EU854490, EU854488, EU854487, EU854484, EU854482, EU854479, DQ011180, DQ011179, DQ011178, DQ011177, DQ011176, DQ011175, DQ011174, DQ011173, DQ011172, DQ011171, DQ011170, DQ011169, DQ011167, DQ011166, DQ011165, AY118166, AY118165, BD437626, BD437615, DQ978981, AY585268, AY585267, AY585266, AY585265, AY585264, AY901981, AY901980, AY901979, AY901978, AY901977, AY901976, AY901975, AY901974, AY901973, AY901972, AY901971, AY901970, AY901969, AY901968, AY901967, AY901966, AY901965, DQ369997, DQ369996, DQ369995, DQ369994, DQ369993, DQ369992, DQ369991, DQ369990, DQ369989, DQ369988, DQ369987, DQ369986, DQ369985, DQ369984, DQ369983, DQ369982, DQ369981, DQ369980, DQ369979, DQ369977, DQ369976, DQ093607, DQ093605, DQ093604, DQ093602, DQ093601, DQ093600, DQ093599, DQ093598, DQ093597, DQ093596, DQ093595, DQ093594, DQ093593, DQ093592, DQ093591, DQ093590, DQ093589, DQ093588, DQ093587, DQ093586, DQ093585, DQ445637, DQ445635, DQ445634, DQ445633, DQ445632, DQ445631, AY463237, AY463236, AY463234, AY463233, AY463232, AY463231, AY463229, AY463228, AY463227, AY463226, AY463225, AY463223, AY463222, AY463221, AY463220, AY463219, AY463217, DQ164129, DQ164127, DQ164126, DQ164122, DQ164121, DQ164119, DQ164118, DQ164117, DQ164116, DQ164115, DQ164114, DQ164113, DQ164112, DQ164111, DQ164110, DQ164108, DQ164107, DQ164106, DQ164105, DQ164104, DQ056418, DQ056417, DQ056416, DQ056415, DQ056414, DQ056413, DQ056412, DQ056411, DQ056410, DQ056409, DQ056408, DQ056406, DQ056405, DQ056404, DQ396399, DQ396397, DQ396396, DQ396395, DQ396394, DQ396393, DQ396391, DQ396390, DQ396389, DQ396388, DQ396387, DQ396386, DQ396385, DQ396384, DQ396383, DQ396382, DQ396381, DQ396380, DQ396379, DQ396378, DQ396377, DQ396376, DQ396375, DQ396374, DQ396373, DQ396372, DQ396371, DQ396370, DQ396369, DQ396368, DQ396367, DQ396366, DQ396365, AY772700, AY772699, AY772698, AY772696, AY772695, AY772694, AY772693, AY772692, AY772690, AY878072, AY878071, AY878070, AY878068, AY878065, AY878064, AY878063, AY878062, AY878061, AY878060, AY878059, AY878058, AY878057, AY878056, AY878055, AY878054, DQ351237, DQ351235, DQ351234, DQ351233, DQ351232, DQ351230, DQ351229, DQ351228, DQ351227, DQ351226, DQ351225, DQ351224, DQ351223, DQ351222, DQ351221, DQ351220, DQ351219, DQ351218, DQ351217, DQ351216, DQ275664, DQ275661, DQ275660, DQ275659, DQ275658, DQ275657, DQ275656, DQ275655, DQ275654, DQ275653, DQ275652, DQ275651, DQ275650, DQ275649, DQ275648, DQ275647, DQ275646, DQ275645, DQ275644, DQ275643, DQ275642, AY703911, AY703910, AY703909, AY703908, FJ199772, FJ199771, FJ199768, FJ199765, FJ199764, FJ199761, FJ199760, FJ199759, FJ199758, FJ199757, FJ199756, FJ199755, FJ199751, FJ199750, FJ199749, FJ199748, FJ199747, FJ199746, FJ199745, FJ199744, FJ199741, FJ199738, FJ199736, FJ199735, FJ199733, FJ199732, FJ199730, FJ199728, FJ199724, FJ199723, FJ199722, FJ199720, FJ199719, FJ199715, FJ199714, FJ199712, FJ199710, FJ199707, FJ199706, FJ199705, FJ199699, FJ199696, FJ199694, FJ199692, FJ199689, FJ199687, FJ199685, FJ199683, FJ199680, FJ199677, FJ199676, FJ199674, FJ199673, FJ199671, FJ199670, FJ199668, FJ199664, FJ199662, FJ199653, FJ199644, FJ199641, FJ199640, FJ199639, FJ199635, FJ199633, FJ199632, FJ199629, FJ199628, FJ199623, FJ199618, FJ199613, FJ199610, FJ199608, FJ199602, FJ199595, FJ199594, FJ199589, FJ199586, FJ199565, FJ199563, FJ199561, FJ199553, FJ199552, FJ199546, FJ199544, FJ199541, FJ199540, FJ199539, FJ199537, FJ199533, FJ199532, AY137008, AY137007, AY137006, AY137005, AY137004, AY137003, AY137002, AY137001, AY137000, AY136999, AY136998, AY136997, AY136996, AY136995, AY136994, AY136993, AY136992, AY136991, AY136990, AY136989, AY136988, AY136987, AY136986, AY136985, AY136984, AY136983, AY136982, AY136981, AY136980, AY136979, AY136978, AY136977, AY136976, AY136975, AY136973, AY136972, AY136971, AY136970, AY136969, AY136968, AY136967, AY136966, AY136965, AY136964, AY136963, AY136962, AY136961, AY136960, AY136959, AY136958, AY136957, AY196517, AY196516, AY196515, AY196514, AY196513, AY196512, AY196511, AY196510, AY196509, AY196508, AY196507, AY196506, AY196505, AY196504, AY196503, AY196502, AY196501, AY196500, AY196499, AY196498, GU253428, GU253426, GU253425, GU253424, GU253423, GU253422, GU253420, GU253419, GU253418, GU253417, GU253416, GU253415, GU253414, GU253413, GU253412, GU253411, GU253410, GU253409, GU253408, GU253407, GU253406, GU253405, GU253404, GU253403, GU253402, GU253401, GU253400, GU253399, GU253397, GU253396, GU253395, EU293450, EU293449, EU293448, EU293447, EU293446, EU293445, EU293444, EF602252, EF602251, EF602250, EF602249, EF602248, EF602247, EF602246, EF602245, EF602244, EF602243, EF602242, EF602241, EF602240, EF602239, EF602238, EF602237, EF602236, EF602233, EF602231, EF602230, EF602229, EF602228, EF602226, EF602225, EF602224, EF602223, EF602222, EF602221, EF602220, EF602219, EF602218, EF602217, EF602215, EF602214, EF602213, EF602212, EF602211, EF602210, EF602209, EF602208, EF602207, EF602206, EF602205, EF602204, EF602203, EF602202, EF602201, EF602200, EF602199, EF602197, EF602196, EF602195, EF602193, EF602192, EF602191, EF602190, EF602189, EF602188, EF602187, EF602186, EF602185, EF602184, EF602183, EF602182, EF602181, EF602180, EF602177, EF602176, EF602174, EF602173, EF602172, AY589937, AY589936, AY589935, AY589934, AY589933, AY589932, AY589931, AY589928, AY589926, AY589925, AY589924, AY589923, AY589922, AY589921, AY589920, AY589919, AY589918, AY589917, AY589916, AY589915, AY589914, AY589913, AY589912, AY589910, AY589909, AY589908, AY589907, AY589906, AY589905, AY589904, AY589903, AY589902, AY589901, AY589900, AY589899, AY589898, AY589897, AY589896, AY589895, AY589894, AY589893, AY589892, AY589891, AY589890, AY589889, AY589888, AY589887, AY589886, AY589885, AY589884, AY589882, AY589881, AY589880, AY589879, AY589877, AY589876, AY589875, AY589874, AY589873, AY589872, AY589870, AF544009, AY228556, AY228557, AY162225, AF411967, AF411966, AF286227, AY043176, AY043175, AY043174, EU152471, EU152470, EU152468, EU152467, EU152466, EU152464, EU152461, EU152459, EU152458, EU152457, EU152455, EU152453, EU152451, EU152450, EU152448, EU152446, EU152445, EU152444, EU152443, EU152442, EU152440, EU152439, EU152438, EU152436, EU152435, EU152433, EU152432, EU152431, EU152430, EU152428, EU152426, EU152425, EU152424, EU152423, EU152421, EU152420, EU152419, EU152418, EU152416, EU152414, EU152411, EU152409

**South Korea**

GQ290728, EF157888

**Spain**

EU255476, EU255374, EU255513, EU255509, GQ241019, GQ241017, GQ241016, GQ241015, GQ240966, GQ240958, GQ241127, GQ241122, GQ241073, DQ878954, GU326187, GU326172, GU326128, GU326122, GU326106, FJ481655, FJ481654, FJ670521, EU884500, EU786681, EU786673, AY017452

**Swaziland**

EU244693, EU244692, EU244691, EU244690, EU244689, EU244688, EU244687, EU244686, EU244685, EU244684, EU244683, EU244682, EU244681, EU244680, EU244679, EU244678, EU244677, EU244676, EU244675, EU244674, EU244673, EU244672, EU244671, EU244670, EU244669, EU244668, EU244667, EU244666, EU244665, EU244664, EU244663, EU244662, EU244661, EU244660, EU244659, EU244658, EU244657, EU244656, EU244655, EU244654, EU244653, EU244652, EU244651, EU244650, EU244649, EU244648, EU244647

**Sweden**

DQ877890, GQ401022, GQ400993, GQ400980, GQ400950, GQ400949, GQ400942, GQ400924, GQ400909, GQ400816, GQ400699, GQ400651, GQ400646, GQ400644, GQ400619, GQ400615, GQ400608, GQ400599, GQ400595, GQ400592, GQ400585, GQ400570, GQ400161, GQ400158, GQ399144, AF394464, AF394462, AF378408, AF378407, AF378405, AF378404, AF378403, AF378402, GU324885, GU324882, GU324879, GU324870, GU324863, GU324862, AY165272, AY165266, AY165260, AY165257, AY165255, AY165253, AY165252, AY165230, AY165226, AY165225, AY165224, AY165223, AY165220, AY165219, AY165216, AY165215, AY165214, AY165213, AY165209, AY165208, AY165207, AY165205, AY165196, AY165187, AY165186

**Switzerland**

GQ848145, GQ848120

**Taiwan**

DQ640290

**Tanzania**

HM572414, HM572412, HM572411, HM572410, HM572409, HM572408, HM572407, HM572406, HM572405, HM572404, HM572403, HM572402, HM572401, HM572400, HM572399, HM572398, HM572397, HM572396, CQ891779, EU251881, EU251871, EU251870, EU251867, EU251863, EU251855, EU251851, EU251849, EU251847, EU251845, EU251842, EU251839, EU251831, EU251828, EU251826, EU251824, EU251814, EU251806, EU251804, EU251803, EU251802, EU251798, EU251792, EU251790, EU251778, EU251776, EU251769, EU251764, EU251763, EU251762, EU251760, EU251758, EU251757, EU251753, EU251749, EU251746, EU251744, EU251742, EU251735, EU251726, EU251721, EU251719, AY253322, AY253321, AY253320, AY253317, AY253313, AY253312, AY253310, AY253308, AY253307, AY253304, AY253303, AF286235, AF286234, AY734560, AY734559, AY734558, AY734556, AY734551, AY734550, AF361875, AF361874

**The Netherlands**

GQ400508, GQ400504, GQ400374, GQ400149, GQ400077, GQ399715, GQ399464, GQ399082

**Ukraine**

DQ055217, DQ055203, DQ055201

**United Kingdom**

DQ879080, DQ879070, FJ653087

**United States of America**

EF195277, DQ009851, EU693892, EU693717, EU693681, DQ465237, AY032091, AY444801, AY444800

**Uruguay**

AY563173, AY563169

**Venezuela**

FJ659642

**Yemen**

AY795906, AY790007, AY790001, AY789999, AY789995, AY789994, AY789992

**Zambia**

AB254156, AB254154, AB254149, AB254148, AB254142, AB254141, FJ496214, FJ496199, FJ496198, FJ496189, AF107381, AF107378, HM120150, HM120149, HM120148, HM120147, HM120146, HM120145, HM120144, HM120143, HM120142, HM120141, HM120140, HM120139, HM120138, HM120137, HM120136, HM120135, HM120134, HM120133, HM120131, HM120130, HM120129, HM120128, HM120127, HM120126, HM120125, HM120124, HM120123, HM120122, HM120121, HM120120, HM120119, HM120118, HM120117, HM120116, HM120115, HM120114, HM120113, HM120112, HM120111, HM120110, HM120109, HM120108, HM120107, HM120106, HM120105, HM120103, HM120102, HM120101, HM120100, HM120099, HM120097, HM120096, HM120095, HM120094, HM120093, HM120092, HM120091, HM120090, HM120088, HM120087, HM120086, HM120085, HM120084, HM120083, HM120082, HM120081, HM120080, HM120079, HM120078, HM120077, HM120075, HM120074, HM120073, HM120072, HM120071, HM120070, HM120069, HM120068, HM120067, HM120066, HM120065, HM120063, HM120062, HM120061, HM120059, HM120058, HM120057, HM120056, HM120055, HM120054, HM120053, HM120051, HM120050, HM120049, HM120047, HM120046, HM120045, HM120044, HM120043, HM120042, HM120041, HM120040, HM120038, HM120037, HM120036, HM120035, HM120034, HM120033, HM120032, HM120031, HM120030, HM120029, HM120028, HM120027, HM120026, HM120025, HM120023, HM120022, HM120021, HM120020, HM120019, HM120018, HM120017, HM120016, HM120015, HM120014, HM120013, HM120012, HM120011, HM120010, HM120009, HM120008, HM120007, HM120006, HM120005, HM120004, HM120003, HM120002, HM120001, HM119999, HM119998, HM119997, HM119996, HM119995, HM119994, HM119993, HM119992, HM119991, HM119990, HM119988, HM119987, HM119986, HM119984, HM119983, HM119982, HM119981, HM119980, HM119979, HM119978, HM119977, HM119976, HM119975, HM119974, HM119972, HM119971, HM119970, HM119969, HM119968, HM119967, HM119966, HM119964, HM119963, HM119962, HM119961, HM119960, HM119959, HM119958, HM119957, HM119956, HM119955, HM119954, HM119953, HM119952, HM119951, HM119950, HM119948, HM119947, HM119946, HM119945, HM119944, HM119943, HM119942, HM119940, HM119939, HM119938, HM119937, HM119936, HM119935, HM119934, HM119933, HM119931, HM119930, HM119929, HM119928, HM119927, HM119926, HM119925, HM119924, HM119923, HM119922, HM119921, HM119920, HM119919, HM119918, HM119917, HM119916, HM119915, HM119914, HM119912, HM119911, HM119910, HM119909, HM119908, HM119907, HM119905, HM119904, HM119903, HM119902, HM119901, HM119899, HM119898, HM119897, HM119896, HM119895, HM119893, HM119892, HM119891, HM119890, HM119889, HM119887, HM119886, HM119885, HM119884, HM119883, HM119882, HM119881, HM119880, HM119879, HM119878, HM119877, HM119876, HM119875, HM119874, HM119873, HM119872, HM119871, HM119870, HM119869, HM119868, HM119867, HM119866, HM119865, HM119864, HM119862, HM119861, HM119860, HM119859, HM119858, HM119857, HM119856, HM119855, HM119854, HM119853, HM119852, HM119851, HM119850, HM119848, HM119847, HM119846, HM119845, HM119844, HM119843, HM119842, HM119841, HM119839, HM119838, HM119836, HM119835, HM119834, HM119833, HM119832, HM119831, HM119830, HM119829, HM119828, HM119827, HM119826, HM119825, HM119824, HM119823, HM119822, HM119821, HM119820, HM119819, HM119818, HM119817, HM119816, HM119815, HM119814, HM119813, HM119812, HM119811, HM119810, HM119809, HM119808, HM119807, HM119806, HM119805, HM119804, HM119803, HM119802, HM119801, HM119800, HM119798, HM119797, HM119796, HM119795, HM119794, HM119793, HM119792, HM119791, HM119790, HM119789, HM119788, HM119787, HM119786, HM119785, HM119784, HM119783, HM119782, HM119781, HM119780, HM119779, HM119778, HM119777, HM119776, HM119775, HM119774, HM119773, HM119772, HM119771, HM119769, HM119768, HM119767, HM119766, HM119765, HM119764, HM119763, HM119762, HM119761, HM119760, HM119759, HM119758, HM119757, HM119756, HM119754, HM119753, HM119752, HM119751, HM119750, HM119749, HM119748, HM119747, HM119746, HM119745, HM119744, HM119743, HM119742, HM119741, HM119740, HM119739, HM119738, HM119737, HM119736, HM119735, HM119734, HM119732, HM119731, HM119730, HM119729, HM119728, HM119727, HM119726, HM119725, HM119724, HM119723, HM119722, HM119721, HM119718, HM119716, HM119715, HM119714, HM119713, HM119712, HM119711, HM119710, HM119709, HM119708, HM119707, HM119706, HM119705, HM119704, HM119703, HM119701, HM119700, HM119698, HM119696, HM119692, HM119691, HM119690, HM119689, HM119688, HM119687, HM119686, HM119684, HM119682, HM119681, HM119680, HM119679, HM119678, HM119677, HM119676, HM119675, HM119674, HM119671, HM119670, HM119669, HM119667, HM119666, HM119665, HM119664, HM119663, HM119662, HM119661, HM119660, HM119659, HM119658, HM119657, HM119656, HM119655, HM119654, HM119653, HM119652, HM119651, HM119650, HM119649, HM119647, HM119646, HM119645, HM119644, HM119643, HM119642, HM119641, HM119640, HM119639, HM119638, HM119637, HM119636, HM119635, HM119634, HM119633, HM119632, HM119631, HM119630, HM119629, HM119628, HM119627, HM119626, HM119625, HM119624, HM119623, HM119622, HM119621, HM119620, HM119619, HM119618, HM119617, HM119616, HM119615, HM119614, HM119613, HM119612, HM119611, HM119610, HM119609, HM119608, HM119607, HM119606, HM119605, HM119604, HM119603, GQ433893, GQ433892, GQ433891, GQ433890, GQ433889, GQ433888, GQ433887, GQ433886, GQ433885, GQ433884, GQ433883, GQ433882, GQ433881, GQ433880, GQ433879, GQ433878, GQ433877, GQ433876, GQ433875, GQ433874, GQ433873, GQ433872, GQ433871, GQ433870, GQ433869, GQ433868, GQ433867, GQ433866, GQ433865, GQ433864, GQ433863, GQ433862, GQ433861, GQ433860, GQ433859, GQ433858, GQ433857, GQ433856, GQ433855, GQ433854, GQ433853, GQ433852, GQ433851, GQ433850, GQ433849, GQ433848, GQ433846, GQ433845, GQ433844, GQ433843, GQ433842, GQ433841, GQ433840, GQ433839, GQ433838, GQ433837, GQ433836, GQ433835, GQ433834, GQ433833, GQ433832, GQ433830, GQ433829, GQ433828, GQ433827, GQ433826, GQ433825, GQ433824, GQ433823, GQ433822, GQ433821, GQ433820, GQ433819, GQ433818, GQ433817, GQ433816, GQ433815, GQ433814, GQ433813, GQ433812, GQ433811, GQ433810, GQ433809, GQ433808, GQ433807, GQ433806, GQ433805, GQ433804, GQ433803, GQ433802, GQ427140, GQ427139, GQ427138, GQ427137, GQ427136, GQ427135, GQ427134, GQ427133, GQ427132, GQ427131, GQ427130, GQ427129, GQ427128, GQ427127, GQ427126, GQ427125, GQ427123, GQ427122, GQ427121, GQ427120, GQ427119, GQ427118, GQ427117, GQ427116, GQ427115, GQ427114, GQ427113, GQ427112, AF286225, AF286224

**Zimbabwe**

FJ445734, FJ445733, FJ445732, FJ445731, FJ445730, FJ445729, FJ445728, AY090856, AY090849, AY090845, FJ445727, FJ445725, FJ445724, FJ445723, FJ445721, FJ445717, FJ445715, FJ445714, FJ445713, FJ445712, FJ445711, FJ445710, FJ445709, FJ445708, FJ445707, FJ445706, FJ445702, FJ445701
