# Supplementary material for: The Origin and Evolutionary History of HIV-1 Subtype C in Senegal
Source: PLoS One. 2012 Mar 28;7(3):e33579. doi: 10.1371/journal.pone.0033579 (PMC3314668; doi:10.1371/journal.pone.0033579)
Supplement: Table S2 — Details of the strains included in the restricted phylogenetic tree analysis from Figures 2 , S2 and S3. (PDF) [file pone.0033579.s006.pdf]

Table S2 : Details of the strains included in the restricted phylogenetic tree analysis from Figure 2, S2 and S3.

STRAIN IDENTIFICATION	ACCESSION NUMBER	COUNTRY CODE	COUNTRY	DATE
1182_48_1229_20030428	DQ877762	BE	Belgium	2003
298224175310447	EU248333	BE	Belgium	2003
728064148815993	EU248453	BE	Belgium	2003
857544175285033	EU248493	BE	Belgium	2003
B1104	AM260271	BI	Burundi	2002
U1305	AM260307	BI	Burundi	2002
393212	AF443083	BW	Botswana	1999
07621	AF443088	BW	Botswana	2000
18802	AF443100	BW	Botswana	2000
2127214	AF443105	BW	Botswana	2000
2210_N	AY829309	BW	Botswana	2001
3920_N	AY829326	BW	Botswana	2001
0067	FR666631	CD	Democratic Republic of Congo	2007
LN082	FJ531453	CN	China	2005
1182_48_4134_20030317	DQ878365	DE	Germany	2003

HR1fNvJNtEjG8lV	GQ400661	DE	Germany	2003
1182_48_1407_20030410	DQ877803	DK	Denmark	2003
1182_48_7101_20030723	DQ878954	ES	Spain	2003
1_304517	EU255374	ES	Spain	2006
PPT42	GQ241073	ES	Spain	2002
PPT91	GQ241122	ES	Spain	2007
PPT96	GQ241127	ES	Spain	2007
H_G_016	AB285757	ET	Ethiopia	2003
H_G_060	AB285777	ET	Ethiopia	2003
H_G_090	AB285792	ET	Ethiopia	2003
H_G_139	AB285808	ET	Ethiopia	2003
H_G_140	AB285809	ET	Ethiopia	2003
H_G_238	AB285833	ET	Ethiopia	2003
H_G_239	AB285834	ET	Ethiopia	2003
H_G_260	AB285843	ET	Ethiopia	2003
288	AY713417	ET	Ethiopia	2002
1182_48_3229_20030523	DQ878136	FR	France	2003
CIRMF22	AY140617	GA	Gabon	1997
404	FN557338	GQ	Equatorial Guinea	2008

1182_48_5096_20030901	DQ878592	GR	Greece	2003
ET1	AY255823	IL	Israel	1999
VL07_103	EU781835	IN	India	2007
CV1199_92_08	GU969550	IT	Italia	2008
R2010	AF457054	KE	Kenya	2000
864766187916044	GQ400273	LU	Luxenburg	2003
PG1	AM071423	MZ	Mozambique	2003
PG17	AM071439	MZ	Mozambique	2003
PG232	AM071443	MZ	Mozambique	2003
MC060	GU199551	MZ	Mozambique	2002
MT001229	GU199564	MZ	Mozambique	2003
692158511671732	GQ399970	NO	Norway	2004
844174932638419	GQ400237	NO	Norway	2003
154002729705766	GQ399069	PT	Portugal	2003
CBR_138	HM102337	PT	Portugal	2008
SU504	AY102486	SD	Sudan	1998
SU254	AY102506	SD	Sudan	1999
20200	AY165187	SE	Sweden	2000
18813	AY165226	SE	Sweden	2000

21063	AY165252	SE	Sweden	2001
21064	AY165253	SE	Sweden	2001
h2C1Zlql7B5bbW0	GQ400651	SE	Sweden	2004
pfvo5PpoMpzcBvi	GQ400816	SE	Sweden	2003
2426_34722	GU324870	SE	Sweden	2006
39HALD	AJ287005	SN	Senegal	1998
142HPD_21_4_99	AJ583715	SN	Senegal	1999
159HALD_14_10_99	AJ583716	SN	Senegal	1999
66HPD_31_7_98	AJ583722	SN	Senegal	1998
86hpd_9_2_99	AJ583739	SN	Senegal	1999
SE_364	AY713416	SN	Senegal	1990
MS002	FM210684	SN	Senegal	2004
MS007	FM210685	SN	Senegal	2004
MS010	FM210686	SN	Senegal	2004
MS011	FM210687	SN	Senegal	2004
MS015	FM210689	SN	Senegal	2004
MS029	FM210691	SN	Senegal	2004
MS245	FM210699	SN	Senegal	2004
MS422	FM210709	SN	Senegal	2004

MS448	FM210712	SN	Senegal	2004
MS475	FM210716	SN	Senegal	2004
MS477	FM210717	SN	Senegal	2004
MS481	FM210718	SN	Senegal	2004
MS487	FM210722	SN	Senegal	2004
MS492	FM210723	SN	Senegal	2004
MS522	FM210725	SN	Senegal	2004
MS540	FM210726	SN	Senegal	2004
MS700	FM210736	SN	Senegal	2004
MS779	FM210737	SN	Senegal	2004
MS816	FM210740	SN	Senegal	2004
MS821	FM210741	SN	Senegal	2004
MS835	FM210745	SN	Senegal	2004
MS855	FM210749	SN	Senegal	2004
MS883	FM210752	SN	Senegal	2004
MS34	FM210753	SN	Senegal	2004
67HPD	FN599718	SN	Senegal	1999
510HALD	FN599737	SN	Senegal	2002
965hald	FN599773	SN	Senegal	2003

980hald	FN599776	SN	Senegal	2003
L065	HE588149	SN	Senegal	2003
2658HALD	HE588150	SN	Senegal	2007
2909HALD	HE588151	SN	Senegal	2007
2911HALD	HE588152	SN	Senegal	2007
2936HALD	HE588153	SN	Senegal	2007
3076HALD	HE588154	SN	Senegal	2007
463HALD	HE588155	SN	Senegal	2006
321HALD	HE588156	SN	Senegal	2003
154HALD	HE588157	SN	Senegal	2003
260HALD	HE588158	SN	Senegal	2002
102HALD	HE588159	SN	Senegal	2000
1189	HE588160	SN	Senegal	1997
1186	HE588161	SN	Senegal	1997
1119	HE588162	SN	Senegal	1997
478HALD	HE588163	SN	Senegal	2002
25Fann	HE588164	SN	Senegal	1997
14Fann	HE588165	SN	Senegal	1997
1083	HE588166	SN	Senegal	1996

SNA3_107	HM002507	SN	Senegal	2007
SNA3_191	HM002515	SN	Senegal	2008
SNA3_220	HM002517	SN	Senegal	2008
SNA3_366	HM002544	SN	Senegal	2009
156	EU244675	SZ	Swaziland	2002
17	EU244681	SZ	Swaziland	2002
013P01	AY136966	ZA	South Africa	2001
033P01	AY136986	ZA	South Africa	2001
036P01	AY136989	ZA	South Africa	2001
042P01	AY136995	ZA	South Africa	2001
DR16	AY589877	ZA	South Africa	2002
DR18	AY589879	ZA	South Africa	2002
DR28	AY589885	ZA	South Africa	2002
DR29	AY589886	ZA	South Africa	2002
DR52	AY589905	ZA	South Africa	2002
DR60	AY589912	ZA	South Africa	2002
DR64	AY589914	ZA	South Africa	2002
SK144B1	AY703911	ZA	South Africa	2004
SK041B1	AY772693	ZA	South Africa	2003

SK011B2	AY901965	ZA	South Africa	2003
SK145B1	AY901976	ZA	South Africa	2004
TV002_patent	BD437626	ZA	South Africa	1998
SK161B1	DQ011170	ZA	South Africa	2004
SK156B1	DQ011171	ZA	South Africa	2004
SK132B1	DQ093594	ZA	South Africa	2004
PS216B1	DQ093600	ZA	South Africa	2004
SK247B1	DQ369994	ZA	South Africa	2005
1795	EF602230	ZA	South Africa	2002
KEH44B	EU152414	ZA	South Africa	2006
CHB398M	EU152435	ZA	South Africa	2006
CHB398B	EU152436	ZA	South Africa	2006
CHB200B	EU152470	ZA	South Africa	2006
2_2R	EU854482	ZA	South Africa	2007
PS178	FJ199738	ZA	South Africa	2004
PS216	FJ199771	ZA	South Africa	2004
CAP255_8w_F1	GQ999988	ZA	South Africa	2005
CAP257_7w_F1	GQ999990	ZA	South Africa	2005
15274	GU253409	ZA	South Africa	2006

17071	GU253410	ZA	South Africa	2006
16923	GU253416	ZA	South Africa	2006
17568	GU253418	ZA	South Africa	2007
KLesa4	GQ433822	ZM	Zambia	2005
KZ2	GQ433840	ZM	Zambia	2005
CP076300055	HM119645	ZM	Zambia	2008
CP076300113	HM119690	ZM	Zambia	2008
CP076300129	HM119705	ZM	Zambia	2008
CP076300132	HM119708	ZM	Zambia	2008
CP076300136	HM119712	ZM	Zambia	2008
CP076300156	HM119731	ZM	Zambia	2008
CP076300164	HM119739	ZM	Zambia	2008
CP076300171	HM119744	ZM	Zambia	2008
CP076300196	HM119767	ZM	Zambia	2008
CP076300201	HM119771	ZM	Zambia	2008
CP076300219	HM119787	ZM	Zambia	2008
CP076300449	HM119870	ZM	Zambia	2008
CP076300471	HM119891	ZM	Zambia	2008
CP076300537	HM119952	ZM	Zambia	2008

CP076300552	HM119962	ZM	Zambia	2008
CP076300598	HM119999	ZM	Zambia	2008
CP076300652	HM120046	ZM	Zambia	2008
CP076300654	HM120047	ZM	Zambia	2008
CP076300667	HM120058	ZM	Zambia	2008
CP076300696	HM120081	ZM	Zambia	2008
CP076302410	HM120110	ZM	Zambia	2008
CP076302487	HM120141	ZM	Zambia	2008
CP076302494	HM120142	ZM	Zambia	2008
TC02_2003	FJ445715	ZW	Zimbabwe	2003
