# Supplementary material for: The Origin and Evolutionary History of HIV-1 Subtype C in Senegal
Source: PLoS One. 2012 Mar 28;7(3):e33579. doi: 10.1371/journal.pone.0033579 (PMC3314668; doi:10.1371/journal.pone.0033579)
Supplement: Table S3 — Dating the subtype C epidemic in general and MSM populations in Senegal. Coalescent based estimations (BEAST) and 95% highest posterior density (HPD) intervals of the MRCA dates and substitution rates of 56 HIV-1 subtype C pol sequences obtained from the general and the MSM population. Results are displayed for all tested substitution rate priors and molecular clock models. (PDF) [file pone.0033579.s007.pdf]

Table S3. Dating the subtype C epidemic in general and MSM populations in Senegal. Coalescent based estimations (BEAST) and 95% highest posterior density (HPD) intervals of the MRCA dates and substitution rates of 56 HIV-1 subtype C *pol* sequences obtained from the general and the MSM population. Results are displayed for all tested substitution rate priors and molecular clock models.

Prior	Molecular clock	Alignment	Marginal likelihood	Bayes Factor (vs. Lognormal)	Substitution rate			tMRCA			tMRCA(MSM)		
					Mean	95%HPD		Mean	95%HPD		Mean	95%HPD	
						Lower	Upper		Lower	Upper		Lower	Upper
U[0,1]	Strict	complete	-6669.19	-6.30	1.59x10^{-3}	1.02x10^{-3}	2.19x10^{-3}	1966	1952	1978	1981	1971	1989
		restricted	-5896.28	-6.61	1.63x10^{-3}	1.06x10^{-3}	2.37x10^{-3}	1966	1950	1979	1981	1971	1989
	Exponential	complete	-6647.07	3.31	1.76x10^{-3}	5.04x10^{-6}	3.47x10^{-3}	1859	1683	1987	1880	1716	1992
		restricted	-5881.06	5.08	1.81x10^{-3}	1.31x10^{-6}	3.84x10^{-3}	1833	1596	1989	1856	1646	1993
	Lognormal	complete	-6654.68	-	1.64x10^{-3}	8.64x10^{-4}	2.44x10^{-3}	1967	1950	1983	1979	1965	1989
		restricted	-5892.76	-	1.68x10^{-3}	8.67x10^{-4}	2.48x10^{-3}	1967	1947	1982	1980	1966	1989
N(2.5x10^{-3},10x10^{-4})	Strict	complete	-6667.72	-6.10	1.69x10^{-3}	1.09x10-^{3}	2.24x10^{-3}	1968	1956	1978	1982	1975	1989
		restricted	-5897.10	-2.85	1.72x10^{-3}	1.17x10^{-3}	2.39x10^{-3}	1969	1956	1980	1982	1974	1989
	Exponential	complete	-6646.83	2.98	2.15x10^{-3}	7.22x10^{-4}	3.77x10^{-3}	1964	1930	1987	1971	1938	1990
		restricted	-5880.82	4.22	2.23x10^{-3}	4.77x10^{-4}	3.65x10^{-3}	1963	1920	1987	1971	1932	1992
	Lognormal	complete	-6653.68	-	1.77x10^{-3}	1.04x10^{-3}	2.46x10^{-3}	1970	1957	1982	1981	1971	1990
		restricted	-5890.53	-	1.81x10^{-3}	1.14x10^{-3}	2.55x10^{-3}	1970	1956	1982	1982	1972	1989
N(2.5x10^{-3},7.5x10^{-4})	Strict	complete	-6669.40	-5.92	1.73x10^{-3}	1.21x10^{-3}	2.22x10^{-3}	1969	1959	1978	1983	1976	1989
		restricted	-5899.39	-3.11	1.80x10^{-3}	1.25x10^{-3}	2.35x10^{-3}	1970	1960	1979	1983	1976	1989
	Exponential	complete	-6647.35	3.66	2.30x10^{-3}	1.05x10^{-3}	3.61x10^{-3}	1972	1949	1986	1978	1956	1991
		restricted	-5883.08	3.98	2.39x10^{-3}	1.15x10^{-3}	3.76x10^{-3}	1972	1951	1987	1978	1960	1992
	Lognormal	complete	-6655.78	-	1.81x10^{-3}	1.20x10^{-3}	2.51x10^{-3}	1971	1959	1982	1982	1972	1989
		restricted	-5892.23	-	1.87x10^{-3}	1.23x10^{-3}	2.57x10^{-3}	1972	1960	1982	1983	1974	1989
N(2.5x10^{-3},5x10^{-4})	Strict	complete	-6670.93	-7.77	1.85x10^{-3}	1.36x10^{-3}	2.37x10^{-3}	1971	1962	1979	1984	1978	1989
		restricted	-5899.32	-3.33	1.91x10^{-3}	1.37x10^{-3}	2.40x10^{-3}	1972	1964	1980	1984	1978	1989
	Exponential	complete	-6645.84	3.13	2.38x10^{-3}	1.50x10^{-3}	3.31x10^{-3}	1975	1963	1986	1981	1968	1989
		restricted	-5882.72	3.88	2.45x10^{-3}	1.42x10^{-3}	3.23x10^{-3}	1975	1962	1986	1981	1969	1990
	Lognormal	complete	-6653.05	-	1.97x10^{-3}	1.31x10^{-3}	2.51x10^{-3}	1974	1964	1982	1983	1976	1989
		restricted	-5891.65	-	2.00x10^{-3}	1.32x10^{-3}	2.53x10^{-3}	1974	1963	1981	1984	1977	1989
